# Changes to Carbon Isotopes in Atmospheric CO_2_ Over the Industrial Era and Into the Future

**DOI:** 10.1029/2019GB006170

**Published:** 2020-11-15

**Authors:** Heather Graven, Ralph F. Keeling, Joeri Rogelj

**Affiliations:** ^1^ Department of Physics Imperial College London London UK; ^2^ Grantham Institute for Climate Change and the Environment Imperial College London London UK; ^3^ Scripps Institution of Oceanography University of California, San Diego La Jolla CA USA; ^4^ ENE Program International Institute for Applied Systems Analysis Laxenburg Austria

**Keywords:** carbon dioxide, radiocarbon, carbon‐13, fossil fuels, nuclear bombs, carbon cycle

## Abstract

In this “Grand Challenges” paper, we review how the carbon isotopic composition of atmospheric CO_2_ has changed since the Industrial Revolution due to human activities and their influence on the natural carbon cycle, and we provide new estimates of possible future changes for a range of scenarios. Emissions of CO_2_ from fossil fuel combustion and land use change reduce the ratio of ^13^C/^12^C in atmospheric CO_2_ (δ^13^CO_2_). This is because ^12^C is preferentially assimilated during photosynthesis and δ^13^C in plant‐derived carbon in terrestrial ecosystems and fossil fuels is lower than atmospheric δ^13^CO_2_. Emissions of CO_2_ from fossil fuel combustion also reduce the ratio of ^14^C/C in atmospheric CO_2_ (Δ^14^CO_2_) because ^14^C is absent in million‐year‐old fossil fuels, which have been stored for much longer than the radioactive decay time of ^14^C. Atmospheric Δ^14^CO_2_ rapidly increased in the 1950s to 1960s because of ^14^C produced during nuclear bomb testing. The resulting trends in δ^13^C and Δ^14^C in atmospheric CO_2_ are influenced not only by these human emissions but also by natural carbon exchanges that mix carbon between the atmosphere and ocean and terrestrial ecosystems. This mixing caused Δ^14^CO_2_ to return toward preindustrial levels in the first few decades after the spike from nuclear testing. More recently, as the bomb ^14^C excess is now mostly well mixed with the decadally overturning carbon reservoirs, fossil fuel emissions have become the main factor driving further decreases in atmospheric Δ^14^CO_2_. For δ^13^CO_2_, in addition to exchanges between reservoirs, the extent to which ^12^C is preferentially assimilated during photosynthesis appears to have increased, slowing down the recent δ^13^CO_2_ trend slightly. A new compilation of ice core and flask δ^13^CO_2_ observations indicates that the decline in δ^13^CO_2_ since the preindustrial period is less than some prior estimates, which may have incorporated artifacts owing to offsets from different laboratories' measurements. Atmospheric observations of δ^13^CO_2_ have been used to investigate carbon fluxes and the functioning of plants, and they are used for comparison with δ^13^C in other materials such as tree rings. Atmospheric observations of Δ^14^CO_2_ have been used to quantify the rate of air‐sea gas exchange and ocean circulation, and the rate of net primary production and the turnover time of carbon in plant material and soils. Atmospheric observations of Δ^14^CO_2_ are also used for comparison with Δ^14^C in other materials in many fields such as archaeology, forensics, and physiology. Another major application is the assessment of regional emissions of CO_2_ from fossil fuel combustion using Δ^14^CO_2_ observations and models. In the future, δ^13^CO_2_ and Δ^14^CO_2_ will continue to change. The sign and magnitude of the changes are mainly determined by global fossil fuel emissions. We present here simulations of future δ^13^CO_2_ and Δ^14^CO_2_ for six scenarios based on the shared socioeconomic pathways (SSPs) from the 6th Coupled Model Intercomparison Project (CMIP6). Applications using atmospheric δ^13^CO_2_ and Δ^14^CO_2_ observations in carbon cycle science and many other fields will be affected by these future changes. We recommend an increased effort toward making coordinated measurements of δ^13^C and Δ^14^C across the Earth System and for further development of isotopic modeling and model‐data analysis tools.

## Key Points

- Carbon isotopes, ^14^C and ^13^C, in atmospheric CO_2_ are changing in response to fossil fuel emissions and other human activities
- Future simulations using different SSPs show continued changes in isotopic ratios that depend on fossil fuel emissions and, for ^13^C, BECCS
- Applications using atmospheric ^14^C and ^13^C in studies of the carbon cycle or other fields will be affected by future changes

## 1 Introduction

Carbon isotopes are present in the atmosphere, ocean, and terrestrial biosphere in ratios of approximately 99% ^12^C/C, 1% ^13^C/C, and 1 × 10^−12 14^C/C. ^12^C and ^13^C are stable isotopes while ^14^C is a radioactive isotope called radiocarbon. Radiocarbon is formed naturally in the upper atmosphere from cosmogenic radiation, which produces neutrons that react with atmospheric nitrogen. Because the isotopic composition of carbon is affected by physical, chemical, and biological processes, these ratios are not constant, and they vary across different carbon pools and over time and space. Precise measurements of small differences in these ratios, together with theoretical or empirical models of isotopic fractionation and mixing, enable the investigation of various aspects of the carbon cycle. Observing and analyzing the changes in carbon isotopic composition of atmospheric CO_2_ can help to understand the natural carbon cycle's response to human activities.

The notation δ^13^C refers to the deviation of the ratio ^13^C/^12^C in a sample from a standard ratio ^13^C/^12^C, referred to as Vienna Pee Dee Belemnite (VPDB). Typical measurement precision is ±0.01–0.03‰ for atmospheric CO_2_. The primary international reference material for δ^13^C is calcite (IAEA‐603 and, formerly, NBS19). Calcite must be converted to CO_2_ to implement the VPDB scale at individual laboratories, which has been shown to result in significant laboratory offsets (WMO/IAEA, 2003). Current activities to address measurement compatibility include the distribution of pure CO_2_ or CO_2_ in whole air reference materials (Brand et al., 2009; Wendeberg et al., 2013; WMO/IAEA, 2018), but achieving long‐term compatibility of δ^13^C measurements in atmospheric CO_2_ made at different laboratories remains a challenge, and laboratory offsets must be considered when compiling data (see section 5).

The notation used for ^14^C is Δ^14^C, which is similar to the definition of δ^13^C in that it refers to deviations from a standard ratio termed “Modern.” The notation Δ^14^C includes a correction for radioactive decay in samples of known age and a correction for mass‐dependent fractionation, defined as Δ in Stuiver and Polach (1977). Assuming that any process discriminating against ^13^C will discriminate approximately twice as strongly against ^14^C, measurements of δ^13^C in a sample can be used to correct for mass‐dependent fractionation. This enables Δ^14^C to uncover effects that are unrelated to simple fractionation processes. Typical measurement precision is ±2–3‰ for atmospheric CO_2_. Reference material used for Δ^14^C measurements is typically oxalic acid (Stuiver, 1983), but whole air reference materials have also been used for atmospheric measurements (Graven et al., 2012b). Whole air and CO_2_ have been used in intercomparisons between radiocarbon laboratories making atmospheric measurements and generally showed compatibility of 2‰ or better (Hammer et al., 2017; Miller et al., 2013), in addition to intercomparison activities using wood cellulose and other materials (e.g., Scott et al., 2010).

In this paper, we review the observed changes in the ^13^C and ^14^C isotopic composition of atmospheric CO_2_ (δ^13^CO_2_ and Δ^14^CO_2_) over the Industrial Period and the processes driving these changes. We review key applications for atmospheric δ^13^CO_2_ and Δ^14^CO_2_ observations from the literature, with an emphasis on global or large‐scale processes. Then we present new simulations of future changes in atmospheric δ^13^CO_2_ and Δ^14^CO_2_ corresponding to future emission scenarios through 2100. We discuss the impacts of these future changes on applications for atmospheric δ^13^CO_2_ and Δ^14^CO_2_ observations and make recommendations for observational and modeling activities for δ^13^C and Δ^14^C.

## 2 The ^14^C and ^13^C Suess Effects

The onset of the Industrial Revolution initiated extensive fossil fuel burning that introduced carbon previously stored in geological reservoirs into the atmosphere. Fossil fuels are completely devoid of ^14^C because they have been stored in geological reservoirs for millions of years, much longer than the ^14^C half‐life of 5,700 years. This gives fossil fuels a Δ^14^C signature of −1,000‰. For ^13^C, the carbon in fossil fuels has an isotopic signature (δ^13^C) that ranges from −44‰ to −19‰ (Andres et al., 2000). The δ^13^C in fossil fuels is lower than the δ^13^C in atmospheric CO_2_ (−8.5‰ to −4‰ from the present through the past 65 million years, Graven et al., 2017; Tipple et al., 2010) because fossil fuel carbon originates from plant materials and the photosynthesis process discriminates against ^13^C. There are also geological processes causing further discrimination against ^13^C for some fossil fuels. There is no fractionation during combustion if combustion is complete, but carbonization can produce fractionation (Turney et al., 2006).

As fossil fuels are slightly depleted in ^13^C and entirely depleted in ^14^C, the burning of fossil fuels increases ^12^CO_2_ at a faster relative rate than ^13^CO_2_ and ^14^CO_2_. This dilution effect, which drives δ^13^C and Δ^14^C downward, is termed “The Suess Effect.” In 1955, Hans Suess published the first observations of ^14^C dilution using tree ring records of atmospheric CO_2_ (Suess, 1955). The “Suess Effect” terminology was also later applied to ^13^C, as the dilution process is similar (Keeling, 1979). Importantly, the decreases observed in atmospheric δ^13^CO_2_ and Δ^14^CO_2_ are governed not only by the amount of fossil fuels burnt but also by other human activities and by natural carbon cycle exchanges and their response to changes in atmospheric composition and climate.

Cement manufacturing also involves “fossil” carbon in that the source material is geological and therefore free of any ^14^C. The source material is carbonate rock, which has a δ^13^C of approximately 0‰. The amount of CO_2_ produced by cement manufacturing is only a few percent of the CO_2_ produced by fossil fuel burning. The global average δ^13^C for all fossil fuel combustion and cement production has been −28‰ to −24‰ (Andres et al., 2016). It has shifted toward more negative values in recent years as the share of combustion from natural gas (δ^13^C ~ −44‰) increases while coal (δ^13^C ~ −24‰) decreases.

Land use changes represent another influence on the carbon cycle from human activities. Land use can have various effects that could impact δ^13^CO_2_ and Δ^14^CO_2_: net transfer of carbon from the biosphere to the atmosphere (or vice versa), changes to the average ^13^C discrimination and its spatial pattern through changes in plant type such as the conversion of forest to pasture, and changes in the residence time of carbon in the biosphere. Overall, land use appears to have had small effects on global mean δ^13^CO_2_ and Δ^14^CO_2_ over the Industrial Period, in part because of responses of natural biospheric and ocean fluxes that compensate for land use effects on δ^13^CO_2_ and Δ^14^CO_2_. However, land use effects could be important regionally and for some applications (Scholze et al., 2008).

## 3 The Nuclear Bomb Effect for ^14^C

In the 1950s and 1960s, nuclear weapons testing produced ^14^C in the atmosphere, strongly enriching ^14^C and counteracting the Suess Effect. This effect was termed the “Atom Bomb Effect” when first reported by Rafter and Fergusson (1957); we refer to it as the “Nuclear Bomb Effect.” The process for ^14^C production was similar to the natural production of ^14^C in the atmosphere: Neutrons produced by the hydrogen bomb explosions react with atmospheric nitrogen to produce ^14^C. Most of the nuclear explosions and ^14^C production took place in the Northern Hemisphere, and most tests and particularly the largest tests occurred shortly before the Partial Test Ban Treaty came into effect in 1963 (Naegler & Levin, 2006).

There is an ongoing production of ^14^C by the nuclear industry at nuclear power plants, with the ^14^C production varying by type of reactor. The amount of ^14^C produced by the nuclear industry and released to the atmosphere is only about 10% of the natural production of ^14^C (Graven & Gruber, 2011), so the effects on Δ^14^CO_2_ are much smaller than the effects from the nuclear weapons testing, which, in contrast, exceeded the rate of natural production by 2 orders of magnitude (Naegler & Levin, 2006). Nuclear power plant emissions ramped up between the 1970s and 1990s as the nuclear industry expanded, but they appear to have recently started to fall (Zazzeri et al., 2018).

## 4 Natural Carbon Cycle Response to the Suess and Nuclear Bomb Effects

**Figure 1:**
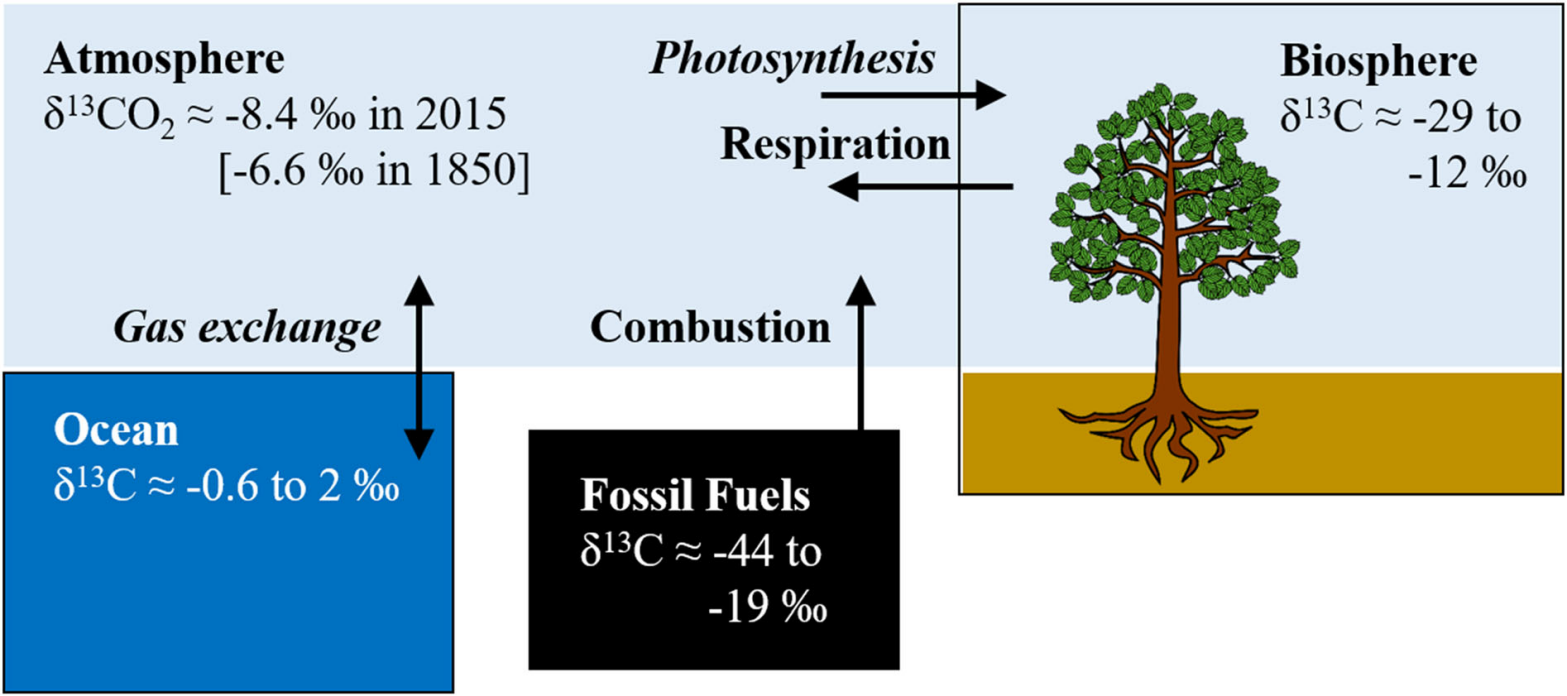
Diagram of ^13^C in the global carbon cycle showing the pools interacting with atmospheric CO_2_ on the timescale of the Industrial Period. Typical ranges of δ^13^C are shown for each of the pools (Andres et al*.*, 2000; Bowling et al*.*, 2008; Graven et al*.*, 2017; Olsen et al*.*, 2019). Global average δ^13^CO_2_ was −8.4‰ in 2015 and −6.6‰ in 1850. Processes involving significant fractionation are shown in italics; processes without significant fractionation are shown in normal text.

**Figure 2:**
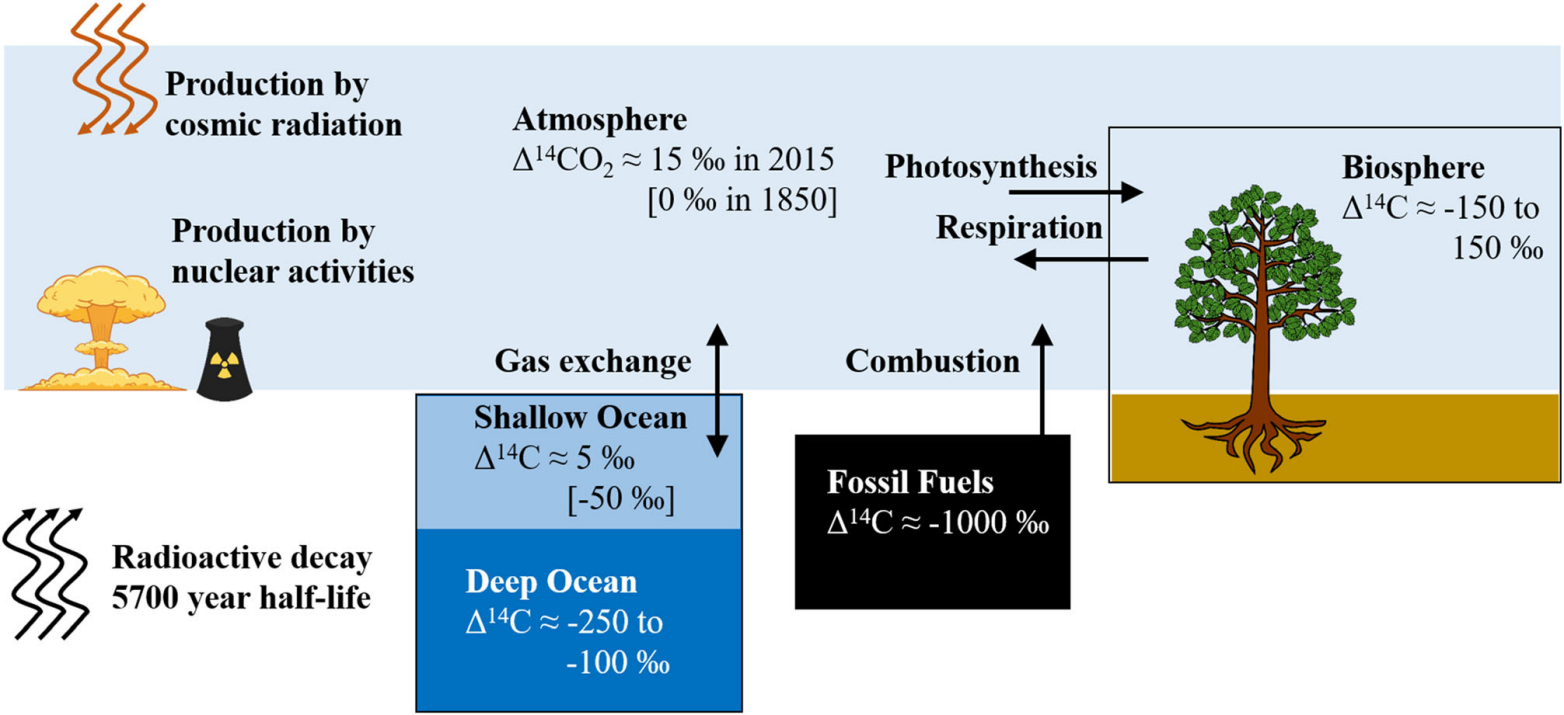
Diagram of ^14^C in the global carbon cycle showing the pools interacting with atmospheric CO_2_ on the timescale of the Industrial Period. Typical ranges of Δ^14^C are shown for each of the pools. Global average Δ^14^CO_2_ was approximately 15‰ in 2015 and 0‰ in 1850, whereas Δ^14^CO_2_ in the troposphere was much higher in 1964–1965, 600‰ to 1,000‰ (Figure 3). In the shallow ocean, average Δ^14^C was approximately 5‰ in 2015 and −50‰ in 1850. Production of ^14^C occurs naturally through cosmic radiation and anthropogenically through nuclear activities. All ^14^C undergoes radioactive decay with a half‐life of 5,700 years.

By perturbing the isotopic composition of atmospheric CO_2_, the Suess and Nuclear Bomb Effects have also affected all the other carbon reservoirs in the ocean and on land that exchange with atmospheric CO_2_ on decadal to centennial timescales (Figures 1 and 2). These exchanges between the atmosphere and other carbon reservoirs have modulated the changes to atmospheric CO_2_, effectively mixing the anthropogenic emissions into a larger carbon pool that encompasses atmospheric CO_2_ and land and ocean carbon with residence times of about a century or less.

On land, the CO_2_ taken up by photosynthesis carries the stable isotopic signature of atmospheric CO_2_, modified by fractionation during photosynthesis (Figure 1). Photosynthetic fractionation, also called discrimination, varies by plant type. Most trees are C_3_ plants that discriminate more than C_4_ plants like grasses, with the δ^13^C of the fixed carbon approximately 18‰ lower in C_3_ and 4‰ lower in C_4_ plants than in atmospheric CO_2_. The CO_2_ returned to the atmosphere by respiration carries the isotopic signature of the organic material being respired, which can have a range of ages. Fractionation does not occur during respiration (Lin & Ehleringer, 1997), although there can be differences in δ^13^C between different plant and soil compounds or gradients within plants that can lead to variation in δ^13^C of respiration (Bowling et al., 2008).

Similarly, the CO_2_ entering the ocean through air‐sea exchange carries the stable isotopic signature of atmospheric CO_2_, modified by fractionation during gas transfer (Figure 2). The CO_2_ exiting the ocean carries the isotopic signature of dissolved inorganic carbon (DIC) at the surface, modified by fractionation during gas transfer. Fractionation during gas transfer includes both kinetic and equilibrium effects (Zhang et al., 1995) and results in ocean DIC being ^13^C‐enriched compared to atmospheric δ^13^CO_2_. The δ^13^C of ocean waters are also influenced by marine ecosystems such that the net photosynthesis in the surface ocean and net respiration at depth cause δ^13^C to generally decrease with depth (Eide et al., 2017).

The gross fluxes to the atmosphere from the terrestrial biosphere and the ocean, and vice versa, also carry the radiocarbon signature of the respective pool. Because of the fractionation correction used in the Δ^14^C notation, the processes involving fractionation do not alter the Δ^14^C signature of the carbon leaving one pool and entering another. Differences in the Δ^14^C signature of different pools are caused by natural or anthropogenic ^14^C production and by radioactive decay. Before the Suess and Nuclear Bomb Effects, Δ^14^C in terrestrial and oceanic pools was lower than atmospheric Δ^14^C because of radioactive decay, depending on how long the carbon was isolated from the atmosphere. The Δ^14^C in new leaves would be nearly the same as atmospheric Δ^14^C, whereas the Δ^14^C in the deep ocean or in aged soils would be much lower.

The decline in atmospheric δ^13^CO_2_ since the Industrial Revolution has resulted in the CO_2_ taken up by photosynthesis being lighter than CO_2_ returned to the atmosphere by respiration. Similarly, the CO_2_ taken up by the ocean is lighter than the CO_2_ returned to the atmosphere. Therefore, the net land exchange and net ocean exchange are causing a net flux of ^13^C from the terrestrial biosphere to the atmosphere and from the ocean to the atmosphere that partly counteracts the decline in atmospheric δ^13^CO_2_. These are referred to as “disequilibrium fluxes.” In addition, the discrimination against ^13^C that occurs during photosynthesis may be increasing over time (Keeling et al., 2017; Schubert & Jahren, 2012), causing even less ^13^C to be removed by photosynthesis. Discrimination is increasing because of the impact of rising atmospheric CO_2_ concentration on photorespiration and mesophyll processes. Individual plants and ecosystems may have also experienced changes in δ^13^C due to variation or trends in climate that influence the strength of ^13^C discrimination. Air‐sea exchange of ^13^C is also influenced by ocean temperature, wind speed, and biological productivity. Changes in these properties may have also caused small influences on the atmospheric δ^13^CO_2_ trend over the Industrial Period (Keeling et al., 2017).

The Suess Effect has a similar effect on ^14^C, such that decreases in atmospheric Δ^14^CO_2_ lead to net effluxes of ^14^C from the land biosphere and the ocean that partly counteract the decrease in atmospheric Δ^14^CO_2_ (Stuiver & Quay, 1981). The nuclear weapons tests had the opposite effect. The Nuclear Bomb Effect caused the atmosphere to become highly enriched in ^14^C and land and ocean exchanges acted to remove ^14^C and decrease Δ^14^CO_2_ (Levin & Hesshaimer, 2000). Now that several decades have passed since the bomb testing ended, the land and ocean exchanges of ^14^C have become more complex. There are both positive and negative influences on Δ^14^CO_2_. Reservoirs where the carbon is stored for a matter of years quickly became more enriched in ^14^C following the atmosphere, but with a lag. Now, as atmospheric Δ^14^C is falling, the Δ^14^C of these reservoirs is again falling behind the atmosphere trend. These reservoirs, which include carbon in terrestrial vegetation and in the surface waters of subtropical ocean gyres, are now positive influences on Δ^14^CO_2_, releasing ^14^C back to the air (Graven, Gruber, et al., 2012; Randerson, Collatz, et al., 2002). Reservoirs that exchange with the atmosphere on longer timescales, such as the carbon in the surface water of the Southern Ocean, remain lower in Δ^14^C and continue to be a negative influence on Δ^14^CO_2_ today (Graven, Gruber, et al., 2012).

In the simple diagrams in Figures 1 and 2, and in the simple carbon cycle model we present later, we have omitted the conduit of terrestrial carbon to the ocean via rivers, which comprises 0.4 to 0.8 PgC year^−1^ (Resplandy et al., 2018). The impacts of rivers on atmospheric δ^13^CO_2_ and Δ^14^CO_2_ are likely to be small overall, since the riverine flux is much smaller than the gross fluxes between atmospheric CO_2_ and the terrestrial biosphere and ocean, but the carbon in rivers will respond to atmospheric δ^13^CO_2_ and Δ^14^CO_2_ and changing environmental conditions that affect terrestrial and riverine carbon cycling. Radiocarbon measurements have revealed differences in the age of dissolved and particulate organic carbon in rivers that help to identify their sources (Marwick et al., 2015). There is also evidence that land use has altered the age of the terrestrial carbon exported to the ocean, where deforestation increases the transport of aged soil organic carbon in rivers and its subsequent remineralization (Drake et al., 2019).

## 5 Atmospheric Changes Over the Industrial Period

The changes in atmospheric δ^13^CO_2_ and Δ^14^CO_2_ over the Industrial Period have been quantified using a combination of direct sampling of the atmosphere and records of atmospheric composition from tree rings, ice cores, and firn. Regular observations of δ^13^CO_2_ and Δ^14^CO_2_ have been made by direct measurements of air samples since the 1970s for δ^13^CO_2_ (Allison & Francey, 2007; Keeling et al., 2005; Vaughn et al., 2010) and the 1950s for Δ^14^CO_2_ (Levin et al., 2010; Turnbull et al., 2016). Records of δ^13^CO_2_ and Δ^14^CO_2_ prior to direct measurements have been constructed using measurements of air in ice cores and firn for δ^13^CO_2_ (Rubino et al., 2013) and tree ring cellulose and other materials for Δ^14^CO_2_ (Hogg et al., 2013; Reimer et al., 2013).

**Figure 3:**
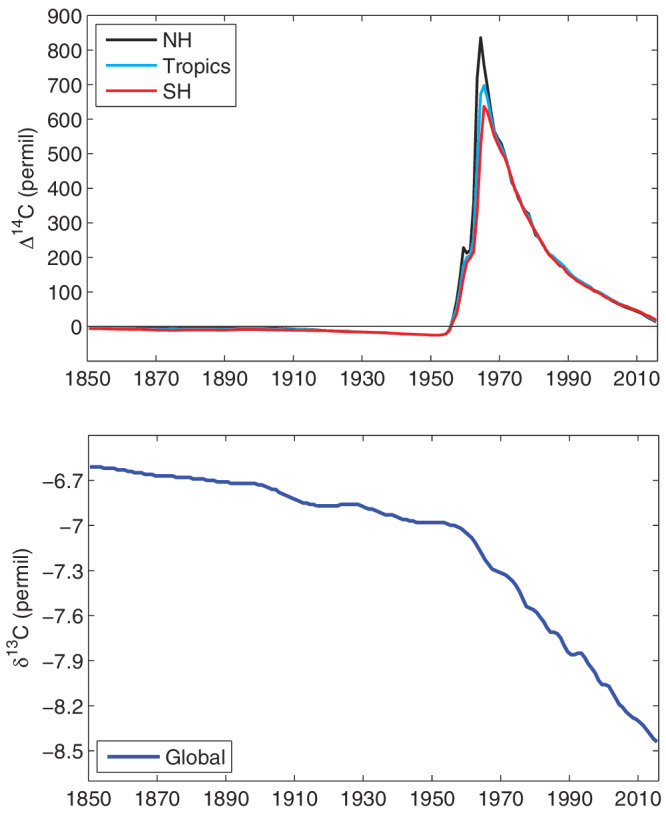
Compiled historical data sets for Δ^14^CO_2_ (top) and δ^13^CO_2_ (bottom) from Graven et al*.* (2017). Annual mean values of Δ^14^C are provided for three zonal bands representing the Northern Hemisphere (30–90°N), the Tropics (30°S to 30°N), and the Southern Hemisphere (30–90°S). Annual mean, global mean values are provided for δ^13^C.

Recently, various records have been compiled and harmonized to provide a consistent record of global δ^13^CO_2_ and Δ^14^CO_2_ changes over the Industrial Period, 1850–2015 (Graven et al., 2017) (Figure 3). These compiled records provide annual averages for global δ^13^CO_2_ and for Δ^14^CO_2_ in three zonal bands.

From 1850 to 2015 atmospheric δ^13^CO_2_ decreased by 1.8‰, with 1.5% of this drop occurring since 1950 (Figure 3) (Graven et al., 2017; Rubino et al., 2013). The Graven et al. (2017) compilation shows a smaller change in δ^13^CO_2_ over the Industrial Period, 1850 to 2015, than in previous estimates. Measurements of δ^13^CO_2_ reported by Bauska et al. (2015) and Friedli et al. (1986) between 1850 and 1950 are approximately 0.05‰ and 0.12‰ higher, respectively, than in Graven et al. (2017) so that when combined with recent flask data the change since 1850 appears larger. The difference arises from the methods to convert calcite ^13^C standards into CO_2_ and implement the VPDB scale at individual laboratories (Brand et al., 2009). Laboratory offsets can be larger than 0.1‰, much larger than the compatibility goal of ±0.01‰ (WMO/IAEA, 2003, 2018). We expect the data reported by Graven et al. (2017) to be the most robust estimate available of the δ^13^CO_2_ change since 1850 because they ensured that the data from both periods were from the same laboratory (CSIRO), while also incorporating recent flask data from other laboratories by quantifying laboratory offsets. Ongoing activities to distribute reference materials of pure CO_2_ or CO_2_ in whole air show promise for improving measurement compatibility (Wendeberg et al., 2013; WMO/IAEA, 2018).

Atmospheric Δ^14^CO_2_ decreased by approximately 20‰ between 1850 and 1950 as a result of fossil fuel emissions after the Industrial Revolution (Suess, 1955) (Figure 3). Then Δ^14^CO_2_ rose rapidly from the mid‐1950s until the mid‐1960s during the period of intense nuclear weapons testing (Rafter & Fergusson, 1957). Tropospheric Δ^14^CO_2_ reached its highest level in 1964–1965, which was 835‰ in the Northern Hemisphere annual average (Figure 3). After the peak in 1964–1965, Δ^14^CO_2_ decreased at a nearly exponential rate as the “bomb ^14^C” mixed into the ocean and terrestrial biosphere. Initially, large gradients were observed between the Northern and Southern Hemispheres because most of the bomb tests occurred in the Northern Hemisphere (Figure 3) (Nydal & Lövseth, 1983). The large interhemispheric gradients in the atmosphere weakened after a few years through atmospheric mixing. Since the 1990s the decrease of Δ^14^CO_2_ has been almost linear at about 5‰ year^−1^, now driven primarily by fossil fuel emissions (Graven et al., 2012b; Levin et al., 2010). The interhemispheric gradient has switched sign: Now Δ^14^CO_2_ in the Northern Hemisphere is about 5‰ lower than in the Southern Hemisphere. Both the Δ^14^CO_2_ trend and the interhemispheric gradient are weaker than expected from fossil fuel emissions alone because of the combined influence on Δ^14^CO_2_ from carbon exchanges with the ocean and land biosphere and by natural ^14^C production and ^14^C emissions from nuclear power plants.

**Figure 4:**
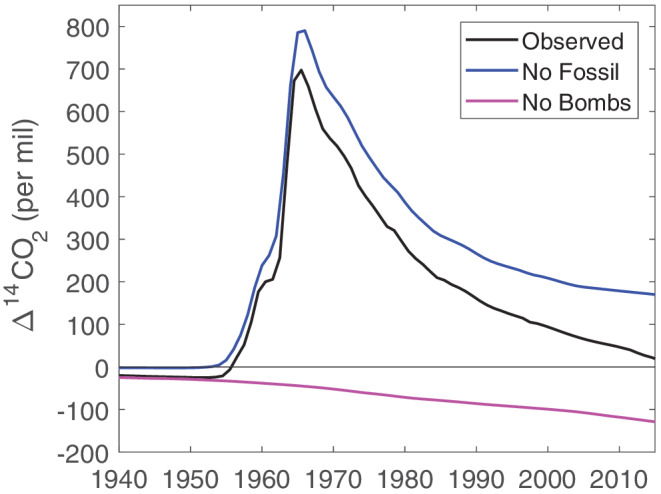
Observed Δ^14^CO_2_ and simulated Δ^14^CO_2_ for scenarios without nuclear weapons tests (“No Bombs”) or without fossil fuel burning (“No Fossil”).

How would atmospheric Δ^14^CO_2_ have evolved in response to the Suess Effect, if there had been no bomb tests? And how would the Nuclear Bomb Effect have evolved in the absence of fossil fuel emissions? To demonstrate the different effects of fossil fuel emissions and nuclear weapons testing on Δ^14^CO_2_, we conducted simulations with a simple carbon cycle model under two hypothetical scenarios (Figure 4). One is a scenario with nuclear weapons testing, but without fossil fuel emissions. The other scenario includes fossil fuel emissions, but no nuclear weapons testing. Details of the simulations are given in Supporting Information Text S1.

Under the scenario without fossil fuel emissions, global atmospheric Δ^14^CO_2_ peaks at a higher level of 790‰ (compared to the observed value in the tropics in 1965 of approximately 700‰) because, in this case, the bomb‐derived ^14^C is mixed into a lower concentration of atmospheric CO_2_ (Figure 4). After the peak in Δ^14^CO_2_, it exponentially declines in a similar way to that observed until around 1990. Then the simulated Δ^14^CO_2_ decline slows, whereas the observed Δ^14^CO_2_ decline continues at a nearly steady rate after 1990. This divergence of the simulated and observed Δ^14^CO_2_ shows how the importance of the Suess Effect has strengthened in the past few decades (Graven et al., 2012b; Levin et al., 2010). Without fossil fuel emissions, Δ^14^CO_2_ would have been about 150‰ higher than observed in 2015.

Under the scenario without nuclear weapons testing, atmospheric Δ^14^CO_2_ decreases throughout the period 1850 to 2015, reaching −130‰ in 2015 (Figure 4). Without the addition of ^14^C from the weapons tests, the Suess Effect would have reduced Δ^14^CO_2_ substantially below preindustrial levels by now.

## 6 Applications of Atmospheric ^13^CO_2_ Measurements

Observations of atmospheric δ^13^CO_2_ have been used in many applications to investigate carbon fluxes and the functioning of plants. A major application has been the so‐called “double deconvolution” on historical CO_2_ and δ^13^CO_2_ data to partition CO_2_ uptake by the ocean versus the terrestrial biosphere (Keeling et al., 1989). These studies use mass balance equations and model simulations that account for fractionation and changing disequilibrium fluxes. The double deconvolution method has been used with direct atmospheric measurements to attribute interannual variations in CO_2_ growth rate to land and ocean sources, concluding that El Niño events are associated with an anomalous terrestrial source of CO_2_ (Keeling et al., 1995). The double deconvolution method has also been used with ice core and firn data to investigate centennial‐ to millennial‐scale variations associated with climate variability, indicating that the terrestrial response to temperature is generally stronger than the ocean's response (Trudinger et al., 1999). The double deconvolution suggested that the low CO_2_ growth rate in the 1940s was driven by the ocean (Trudinger et al., 2002), although this conclusion remains controversial (Bastos et al., 2016; Rafelski et al., 2009).

Atmospheric inversions have been used to estimate spatially resolved fluxes of carbon and ^13^C based on atmospheric data and models. These operate similarly to the double deconvolution. The first study employed a two‐dimensional atmospheric model and helped to identify the “missing sink” of carbon in the land biosphere and particularly in the Northern Hemisphere (Ciais et al., 1995). Subsequent three‐dimensional studies indicated that land and ocean CO_2_ sinks were comparable in magnitude and that CO_2_ uptake increased in the Northern Hemisphere after the Pinatubo eruption in 1991, in addition to the interannual variability related to El Niño (Enting et al., 1995; Rayner et al., 1999). A shortcoming of these studies was that variability in plant ^13^C discrimination was not considered. In reality, plant ^13^C discrimination and CO_2_ uptake are expected to be correlated, for example, because drought will reduce both productivity and discrimination as plants close their stomata to minimize water loss (Randerson, Enting et al., 2002). Expanding the methodology to estimate discrimination as part of the inversion, Peters et al. (2018) estimated variations in water use efficiency on continental scales and showed that global models underestimated the drought response of plants.

The potential for long‐term trends in plant discrimination had also been neglected in global studies using the double deconvolution. Using historical δ^13^CO_2_ data with a simple carbon cycle model, Keeling et al. (2017) found that ^13^C discrimination is likely to have strengthened by 0.7‰ between 1975 and 2005, which is consistent with a dependence on CO_2_ concentration that has been found in laboratory and paleo studies and attributed to mesophyll and photorespiration effects (Schubert & Jahren, 2012). Keeling et al. (2017) further argue that the past double deconvolution studies have neglected a mechanistic link between land and ocean isotopic fluxes that means long‐term δ^13^CO_2_ data actually do not provide a strong constraint on land and ocean CO_2_ sinks. For example, changing the ocean diffusivity in a simple model changes the ocean CO_2_ uptake and ^13^C flux, but it creates compensating changes in the ^13^C flux to the land via the residual CO_2_ flux needed to maintain mass balance. Therefore, ocean diffusivity (which governs ocean CO_2_ uptake) does not have a strong influence on the long‐term δ^13^CO_2_ trend.

Atmospheric δ^13^CO_2_ measurements are commonly used to investigate terrestrial biosphere activity on local or regional scales by estimating isotopic signatures of photosynthesis or respiration using the “Keeling Plot” approach. The “Keeling Plot” (Keeling, 1958), or alternative formulations such as the “Miller‐Tans Plot” (Miller & Tans, 2003), quantifies the isotopic signature of a CO_2_ source or sink by manipulating the CO_2_ and ^13^CO_2_ mass balance equations so that the isotopic signature is given by the intercept or slope of a regression fit. These studies have revealed a strong link between isotopic fluxes and water availability (Pataki et al., 2003). They have helped to explain the driving factors of water use efficiency by plants, a metric for the amount of productivity per unit water loss, and how these factors affect spatial and temporal patterns of water use efficiency (Bowling et al., 2002). These studies typically sample air in flasks that are subsequently analyzed for δ^13^CO_2_ by mass spectrometry in the laboratory, but now optical instruments that measure ^13^CO_2_ are increasingly used in the field. These instruments have also enabled eddy covariance measurements of ^13^CO_2_ fluxes, uncovering the suppression of daytime respiration (Wehr et al., 2016).

Other studies have measured δ^13^CO_2_ in urban areas to investigate fossil fuel emissions. In combination with other tracers such as Δ^14^CO_2_ or δ^18^O of CO_2_, δ^13^CO_2_ measurements have been useful for determining the proportion of natural gas versus petroleum contributions to fossil fuel CO_2_ emissions in urban areas (Newman et al., 2016; Pataki et al., 2007).

Measurements of atmospheric δ^13^CO_2_ are also critical to other studies that do not interpret the measurements directly but rather use them for comparison with δ^13^C measured in other materials. In terrestrial ecology, atmospheric δ^13^CO_2_ is compared to δ^13^C in tree rings or leaves to investigate spatial patterns and temporal variation in the internal leaf CO_2_ concentration and thereby, the response of plant productivity to climate, atmospheric CO_2_, and other variables (Frank et al., 2015; Wang et al., 2017). Measurements of δ^13^C in DIC in the ocean have been compared with atmospheric δ^13^CO_2_ to estimate anthropogenic CO_2_ uptake (Gruber & Keeling, 2001; Quay et al., 2003). Comparisons with atmospheric δ^13^CO_2_ are also used in ecological studies of the diet, trophic structure, physiology, and local environment of animals (DeNiro & Epstein, 1978).

## 7 Applications of Atmospheric ^14^CO_2_ Measurements

Observations of atmospheric Δ^14^CO_2_ have been used in many applications to investigate the global carbon cycle (Levin & Hesshaimer, 2000). The Suess (1955) measurement of industrial‐era Δ^14^CO_2_ via tree ring records comprised some of the first evidence of the strong impact of fossil fuel burning on atmospheric CO_2_, predating the start of C. D. Keeling's long‐term CO_2_ concentration measurements (Keeling, 1960). The first direct measurements of atmospheric Δ^14^CO_2_ were made around the same time as the nuclear weapons tests, revealing large spatial gradients caused by the location of the nuclear tests. These observations were used to investigate atmospheric mixing and showed that the interhemispheric exchange time in the troposphere is about 1 year and the mixing between the stratosphere and troposphere has a seasonal variation (Lal & Rama, 1966; Nydal, 1966).

Other studies have investigated ocean or terrestrial biosphere CO_2_ fluxes using Δ^14^CO_2_ measurements. By using Δ^14^CO_2_ measurements and carbon cycle models to construct an inventory of bomb‐derived ^14^C in each of the main carbon reservoirs, Hesshaimer et al. (1994) showed that previous estimates of the ocean ^14^C inventory (Broecker et al., 1985) had been too high. This implied that the depth to which bomb‐derived ^14^C had penetrated into the ocean and the amount of CO_2_ that had been taken up were also overestimated. Several other studies have used oceanic measurements of Δ^14^C in DIC to estimate the air‐sea gas exchange velocity (Naegler et al., 2006; Sweeney et al., 2007; Wanninkhof, 2014). Changes in ocean circulation that impact the air‐sea exchange of ^14^C have been inferred from Δ^14^CO_2_ measured on timescales of interannual, El Niño events (Rozanski et al., 1995) and timescales of decades to centuries (Rodgers et al., 2011). The magnitude of net primary production in the terrestrial biosphere has also been estimated (Naegler & Levin, 2009) using Δ^14^CO_2_ measurements and carbon cycle models to construct an inventory of bomb‐derived ^14^C, in a similar approach to Hesshaimer et al. (1994). A few studies have also considered the effect of biospheric carbon fluxes on atmospheric Δ^14^CO_2_ measurements. Signatures of elevated Δ^14^C in respiration were postulated for seasonal cycles of Δ^14^CO_2_ in North America (LaFranchi et al., 2016) and for the large‐scale meridional gradients of Δ^14^CO_2_ (Levin & Hesshaimer, 2000).

A major and growing application for atmospheric Δ^14^CO_2_ measurements is the calculation of local CO_2_ added by fossil fuel combustion (ffCO_2_). Evidence for a regional Suess Effect had already appeared in comparisons of tree ring data (Tans et al., 1979). Then I. Levin developed the methodology for the calculation of ffCO_2_ with atmospheric observations in Europe in the 1980s (Levin et al., 1989). The method attributes regional gradients in Δ^14^CO_2_ to fossil fuel emissions, while accounting for other regional influences on Δ^14^CO_2_ from heterotrophic respiration and nuclear power plants (*β*) (Turnbull et al., 2006):

(1) $$\mathrm{ffC}O_{2}=C_{m}\frac{\Delta \mathrm{bg}-\Delta m}{\Delta \mathrm{bg}+1,000‰}+\beta$$

Here *C*_*m*_ is the measured CO_2_ concentration, Δ*m* is the measured Δ^14^CO_2_, and Δ*bg* is the Δ^14^CO_2_ at a “background” site that is upwind of the region of interest. *β* represents a correction for nonfossil fuel influences on Δ^14^CO_2_, which could include heterotrophic respiration or ^14^C emissions from nuclear power plants. I. Levin and colleagues have measured Δ^14^CO_2_ in the city of Heidelberg since 1986, comparing it to measurements from Jungfraujoch in the Swiss Alps to calculate ffCO_2_ (Levin et al., 2003, 2011). Their measurements have shown little change in the ffCO_2_ present in Heidelberg, similar to reported trends in local emissions. Observing system simulation experiments have demonstrated that Δ^14^CO_2_ measurements have a strong potential for improving atmospheric observation‐based estimates of not only regional fossil fuel emissions but also biospheric fluxes (Basu et al., 2016; Fischer et al., 2017). In the state of California, USA, measurements of Δ^14^CO_2_ from a network of towers were combined with a regional atmospheric transport model in an atmospheric inversion to estimate fossil fuel emissions, finding that reported emissions were consistent with Δ^14^CO_2_ observations (Graven et al., 2018). In an atmospheric inversion applied to Δ^14^CO_2_ measurements across North America, estimated emissions for the entire United States were consistent with those officially reported but significantly higher than some other commonly used fossil fuel emissions data products (Basu et al., 2020). Some other studies have combined Δ^14^CO_2_ measurements with CO, a combustion product that can be measured continuously (Turnbull et al., 2015; Vogel et al., 2010).

Applications making use of Δ^14^CO_2_ measurements for comparison with Δ^14^C in other materials are much more numerous than for δ^13^CO_2_, and they span a broad range of fields including archaeology, physiology, and forensics (Bronk Ramsey, 2008; Geyh, 2001; Spalding et al., 2005). Within carbon cycle science, Δ^14^C measurements are widely used in ecology and soil science to determine the residence time of carbon in different compound classes (Trumbore, 2000).

Some applications combine δ^13^C and Δ^14^C to draw more powerful inferences from the combination that was possible with either alone. For example, Keeling et al. (2017) showed that atmospheric δ^13^CO_2_ trends could not be matched by a carbon cycle model constrained by radiocarbon data, unless changes in ^13^C discrimination during photosynthesis were included in the model. Krakauer et al. (2006) analyzed spatial patterns in both atmospheric Δ^14^CO_2_ and δ^13^CO_2_ to investigate the air‐sea gas exchange velocity.

## 8 Projected Future Changes in δ^13^CO_2_ and Δ^14^CO_2_

In the future, atmospheric δ^13^CO_2_ and Δ^14^CO_2_ will continue to evolve in response to fossil fuel emissions and other human activities, and the carbon cycle responses to them. Future simulations of Δ^14^CO_2_ were first presented by Caldeira et al. (1998) for the IS92a “business‐as‐usual” emission scenario from the first IPCC Assessment Report. They showed that increasing fossil fuel emissions cause Δ^14^CO_2_ to decrease to lower than −150‰ in 2100. While Δ^14^CO_2_ decreases strongly, the number of atoms of ^14^C in the atmosphere actually increases due to a large efflux of ^14^C from the ocean to the atmosphere in response to the changing air‐sea disequilibrium. Graven (2015) ran similar simulations using the Representative Concentration Pathways from the fifth IPCC Report considering not just business‐as‐usual but a range of future scenarios (Meinshausen et al., 2011). She found a range of possible paths for Δ^14^CO_2_ through this century, with the high fossil fuel emission scenario dropping to less than −230‰ in 2100 but a mitigation scenario in line with limiting global warming below 2°C dropping to about −20‰ in the 2030s and then remaining nearly steady. She made important inferences about the impacts of these different scenarios. The high fossil fuel emission scenario creates ambiguity in the use of radiocarbon dating because at some point during the century “new” materials would have the same radiocarbon age as materials that are up to 2,000 years old, with impacts on archaeology and forgery detection. In contrast, scenarios where Δ^14^CO_2_ stops decreasing imply that applications in ecology, forensics, and physiology that make use of the Δ^14^CO_2_ trend as a shorter‐term clock would no longer be viable.

The first simulations of future δ^13^CO_2_ were presented by Köhler (2016) using the Representative Concentration Pathways. They showed continued declines in δ^13^CO_2_ as fossil fuel emissions grow in high‐emission scenarios, but reversals of δ^13^CO_2_ trends for low‐emission scenarios. There was a range of about 5‰ between the high fossil fuel emission and mitigation scenarios in 2100, with the most stringent mitigation scenario reaching a minimum around midcentury and then increasing by several per mil.

**Figure 5:**
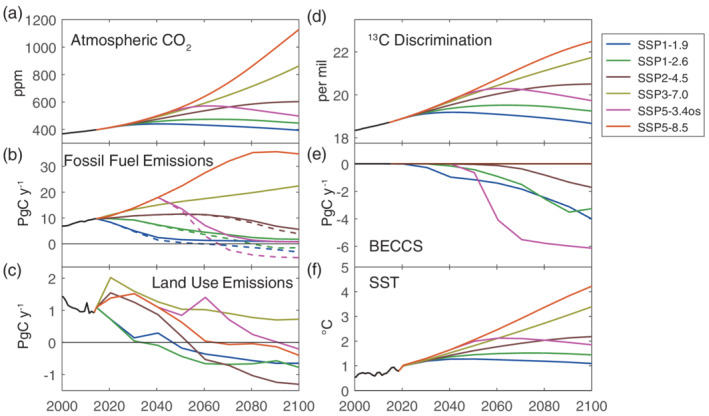
(a) Atmospheric CO_2_, (b) fossil fuel emissions, (c) land use emissions, (d) ^13^C discrimination, (e) CO_2_ removal by BECCS, and (f) global mean sea surface temperature (SST) used in the future simulations. In (b) the gross fossil fuel emissions are shown with solid lines while dashed lines show net emissions accounting for BECCS. Historical data are shown in black until 2015, and then the six SSP‐based scenario projections are shown for 2015–2100.

The future scenarios being considered for the sixth IPCC Report by the Coupled Model Intercomparison Project (CMIP) are now based on a set of five narratives, called the shared socioeconomic pathways (SSPs) (O'Neill et al., 2014). Scenarios ranging from worlds without climate action to very stringent mitigation scenarios in line with limiting global warming to 1.5°C have been explored for each of these narratives (Riahi et al., 2017; Rogelj et al., 2018). Finally, a selection of SSP‐based scenarios have been identified as the main scenarios to be examined in CMIP6 (O'Neill et al., 2016). The atmospheric CO_2_ concentration, fossil fuel emissions, and land use emissions for six of the key SSP‐based scenarios are shown in Figure 5 (Hoesly et al., 2018; Meinshausen et al., 2017). These pathways employ varying amounts of “negative emissions” from deliberate CO_2_ removal, and the net fossil fuel emissions including negative emissions are also shown in Figure 5. These SSP‐based scenarios span a larger range of possible future pathways than the RCPs, including a lower‐emission pathway consistent with a maximum end‐of‐century warming of 1.5°C (SSP1‐1.9) as well as a very high emission pathway without controls on greenhouse gas emissions (SSP5‐8.5). There is also an “overshoot” scenario where atmospheric CO_2_ concentration rises until midcentury and then decreases rapidly as a result of strong and targeted CO_2_ removal activities (SSP5‐3.4os). The process for deliberate CO_2_ removal included in the SSP scenarios is Bioenergy with Carbon Capture and Storage (BECCS). In this way, the CO_2_ removal is mediated by an initial uptake into the terrestrial biosphere, which has implications for atmospheric δ^13^CO_2_ (Köhler, 2016). BECCS acts like an “anti‐Suess Effect,” enriching atmospheric δ^13^CO_2_ by preferentially removing ^12^C through photosynthesis and burial of biofuel‐derived CO_2_.

We conduct simulations of future atmospheric δ^13^CO_2_ and Δ^14^CO_2_ that consider the change in atmospheric CO_2_ concentration, fossil fuel emissions, land use emissions, and BECCS, as well as the response of the carbon cycle to these changes for these six SSP‐based scenarios (Figure 5). In addition, future changes in ^13^C discrimination by land plants are included as a function of atmospheric CO_2_ concentration following Schubert and Jahren (2015), and changes in air‐sea fractionation factors are included as a function of sea surface temperature and dissolved carbonate concentration (Orr et al., 2017). Future changes to the δ^13^C in fossil fuel emissions were not included because there was not enough information provided with the SSP‐based scenarios to estimate them. Further details of the future simulations are given in Text S2.

**Figure 6:**
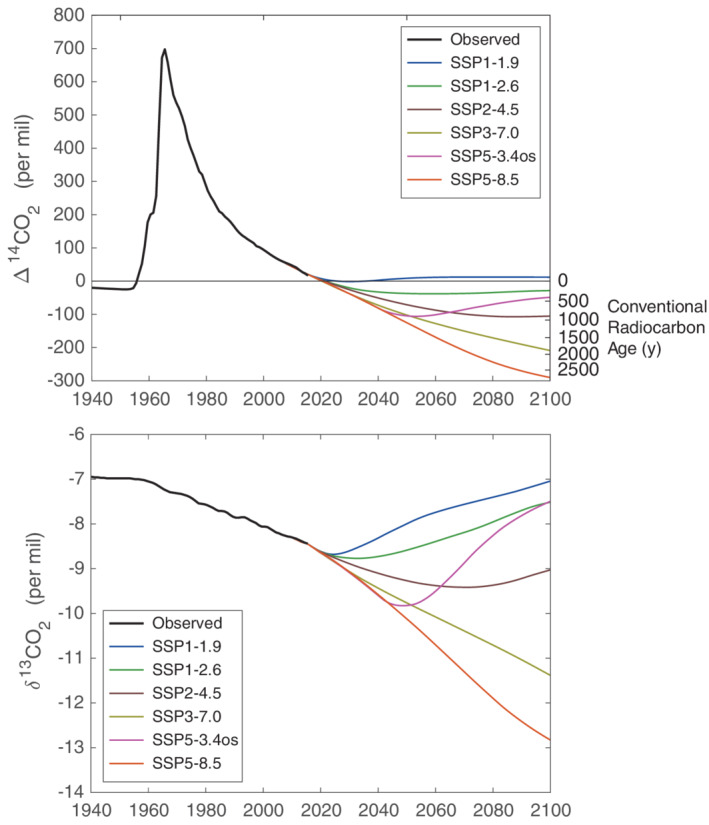
Observed Δ^14^CO_2_ and δ^13^CO_2_ for 1940 to 2015 and simulated Δ^14^CO_2_ and δ^13^CO_2_ for 2015 to 2100 for the six SSP‐based CMIP6 ScenarioMIP scenarios. Colored lines show the midrange values across the 32 sets of parameters used in the simulations. The right axis in the top panel shows the conventional radiocarbon age of a carbon‐containing specimen with the same radiocarbon content, calculated by 8,033 * ln(Δ^14^C/1,000‰ + 1).

The simulations show that atmospheric Δ^14^CO_2_ drops below 0‰ within the next few years in all scenarios (Figure 6). In the lowest emission scenario, SSP1‐1.9, where net fossil fuel emissions reach zero around 2050 (Figure 5), Δ^14^CO_2_ stays around 0‰ for about 10 years and then increases again, remaining at about 10–12‰ for the second half of the century. In this scenario, the effect of a small amount of continued fossil fuel emissions is roughly balanced by other ^14^C fluxes. The less ambitious mitigation scenario SSP1‐2.6 reaches a minimum of −38‰ in the 2050s and then rebounds slightly. The simulated Δ^14^CO_2_ for SSP1‐2.6 is approximately 20‰ lower than the simulated Δ^14^CO_2_ for RCP2.6 in Graven (2015) due to the different structure of the model biosphere, the different criteria for selecting model parameters, and differences between emissions in SSP1‐2.6 and RCP2.6 (see Text S2).

The scenarios SSP2‐4.5, SSP3‐7.0, and SSP5‐8.5 include the least mitigation of emissions and simulated Δ^14^CO_2_ declines steadily until late in the century. Atmospheric Δ^14^CO_2_ reaches −105‰, −209‰, and −290‰ for SSP2‐4.5, SSP3‐7.0, and SSP5‐8.5, respectively. SSP2‐4.5 and SSP3‐7.0 are comparable to RCP4.5 and RCP8.5, which were simulated to reach −80‰ and −254‰ by Graven (2015). In this case the differences in the model structure, calibration, and scenario cause Δ^14^CO_2_ to be 25‰ lower or 40‰ higher Δ^14^CO_2_ in 2100. The scenario SSP5‐8.5 has stronger emissions than any of the RCPs and therefore a more negative Δ^14^CO_2_ in 2100.

In the overshoot scenario SSP5‐3.4os, Δ^14^CO_2_ is simulated to rebound quickly after 2050 due to the reduction in fossil fuel emissions and the rapid implementation of BECCS. The input of fossil carbon is rapidly reduced, and the removal of lower‐Δ^14^C carbon, relative to the carbon in the shallow ocean and terrestrial biosphere, leads to a net efflux of ^14^C back to the atmosphere that increases Δ^14^CO_2_.

Simulated δ^13^CO_2_ declines to approximately −8.7‰ in 2025 in all SSP‐based scenarios and then diverges. All scenarios that we explored that reach a peak and then reduce fossil fuel emissions (all but SSP3‐7.0 and SSP5‐8.5) show an inflection in δ^13^CO_2_ that is more pronounced than for Δ^14^CO_2_. In SSP1‐1.9, δ^13^CO_2_ is approximately −7‰ in 2100, about the same as it was in 1940. SSP1‐2.6 and SSP5‐3.4os both have δ^13^CO_2_ of approximately −7.5‰ in 2100, after a stronger decline and reversal in SSP5‐3.4os compared to SSP1‐2.6. SSP2‐4.5 reaches a minimum of −9.4‰ in 2070 and then returns to −9‰ by 2100. SSP3‐7.0 and SSP5‐8.5 decrease through the century and reach −11.4‰ and −12.8‰, respectively, in 2100.

**Figure 7:**
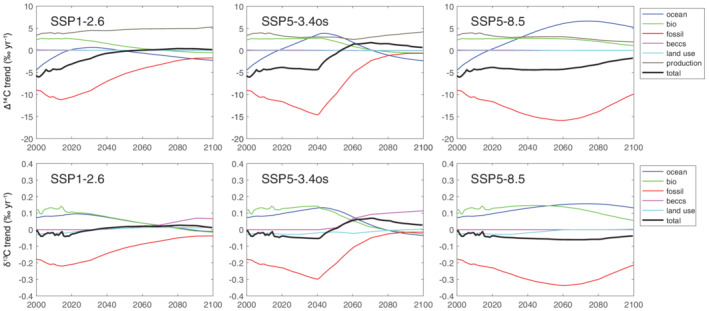
Simulated trend components for Δ^14^CO_2_ (top row) and δ^13^CO_2_ (bottom row) for SSP1‐2.6, SSP5‐3.4os, and SSP5‐8.5. Other SSP‐based scenarios are shown in Figure S1. Colored lines show the midrange values across the 32 sets of parameters used in the simulations. BECCS and land use contributions are uniformly near zero for Δ^14^CO_2_.

In Figure 7, we show the individual contributions to the trends in δ^13^CO_2_ and Δ^14^CO_2_ for SSP1‐2.6, SSP5‐3.4os, and SSP5‐8.5. The contributions for SSP1‐1.9, SSP2‐4.5, and SSP3‐7.0 are shown in Figure S1. Over the recent past, 2000–2015, the negative influence of the ocean weakens from about −5‰ year^−1^ to zero while the negative influence of fossil fuel emissions strengthens slightly. Positive influences from biospheric exchange and from ^14^C production by natural cosmogenic radiation and by nuclear power plants have relatively constant positive influences of 3–4‰ year^−1^ over 2000–2015. Trend contributions of similar magnitudes were found in the early 2000s by Levin et al. (2010) and Graven et al. (2012b).

After 2015 the SSP scenarios diverge. The fossil fuel influence weakens in SSP1‐2.6, followed by an inflection in the oceanic and biospheric contributions, which turn negative around midcentury. After this point, the positive influence of ^14^C production is approximately balanced by the other influences, and Δ^14^CO_2_ remains around −30‰ (Figure 6). In these simulations, nuclear power plant ^14^C emissions are assumed to stay constant at 2008 values throughout 2100. In reality, these emissions could increase or decrease, depending on the future changes in the nuclear industry. However, nuclear power plant ^14^C emissions are only about 10% of natural ^14^C production, so their changes are unlikely to have a large impact on these simulations. Even though natural and nuclear power plant ^14^C production is constant in the future simulations, its contribution to the Δ^14^CO_2_ trend varies over time and across different simulations because it depends on the CO_2_ concentration in the atmosphere. BECCS and land use have essentially no effect on Δ^14^CO_2_ in SSP1‐2.6 or in any other SSP‐based scenario assessed here.

For SSP5‐3.4os and SSP5‐8.5, the negative influence of fossil fuel emissions strengthens until 2040 (SSP5‐3.4os) or 2060 (SSP5‐8.5). Over this time, the overall trend in Δ^14^CO_2_ remains approximately steady at −4‰ year^−1^, which results from the change in oceanic influence counteracting the strengthening in fossil fuel influence. Other influences remain steady. In 2040, SSP5‐3.4os begins a rapid reduction in fossil fuel emissions (Figure 5). About 10 years later, Δ^14^CO_2_ starts to increase. The rapid weakening of the fossil fuel influence on Δ^14^CO_2_ leads to a net positive trend in Δ^14^CO_2_ starting in the 2050s. This suggests that a rapid decarbonization of the energy system could lead to the first increase in Δ^14^CO_2_ since the “bomb peak” in 1964–1965. In contrast, the decreasing trend in SSP5‐8.5 remains remarkably steady before weakening in the last few decades of the century. In this scenario, the positive influence of ocean exchange becomes twice as strong as the positive influence of ^14^C production. The strong positive influence from the ocean is a major reversal from the preindustrial period when the ocean was the main negative influence counteracting natural ^14^C production and from the 20th century period when the ocean was the main sink for bomb ^14^C.

In all scenarios, the biospheric influence responds to the change in the atmospheric trend that governs the biospheric disequilibrium. For example, as the Δ^14^CO_2_ trend slows, the biospheric disequilibrium weakens because Δ^14^C of previously assimilated carbon is more similar to present Δ^14^CO_2_. Sign changes in the Δ^14^CO_2_ trend lead to sign changes in the biospheric disequilibrium and influence on the Δ^14^CO_2_ trend. The effect is modulated by the turnover time of carbon in the biosphere, between 36 and 56 years (Text S2, Naegler & Levin, 2009). The oceanic influence responds to changes in the Δ^14^CO_2_ trend with a longer effective turnover time and with exchanges between many vertical boxes.

For δ^13^CO_2_, the positive biospheric and oceanic contributions to the trend nearly balance the negative fossil fuel contribution over 2000–2015. The negative influence from land use is much smaller and similar to the overall trend. In SSP1‐2.6 fossil fuel emissions peak and slowly weaken after 2015, leading to a weaker negative trend in δ^13^CO_2_. Interestingly, the fossil fuel emissions in 2030 are not much smaller than 2015, but the overall trend in δ^13^CO_2_ is positive. The weakening in the biospheric and oceanic contributions happens more slowly than for the fossil fuel contribution, resulting in an overall positive trend in δ^13^CO_2_ despite the continued fossil fuel emissions. This indicates the negative trend in δ^13^CO_2_ that has been taken as an indication of the ^13^C Suess Effect is actually dependent not just on the presence of fossil fuel emissions but on the acceleration in fossil fuel emissions or their magnitude. In SSP1‐2.6 the effect of land use switches sign around 2030 but remains small. After 2070, BECCS is the strongest positive contribution to the δ^13^CO_2_ trend. The disequilibria in the biosphere and ocean switch sign around 2080 after several decades of increasing δ^13^CO_2_.

In SSP5‐3.4os, the patterns are similar to SSP1‐2.6 but more extreme as a result of the rapid drop in fossil fuel emissions and the rapid expansion of BECCS. Between 2040 and 2060, the δ^13^CO_2_ trend changes from about −0.05‰ year^−1^ to more than + 0.05‰ year^−1^. In SSP5‐8.5, δ^13^CO_2_ continues to decrease even after fossil fuel emissions growth stalls in 2080, unlike the other two SSPs where the δ^13^CO_2_ trend turned positive after fossil fuel emissions weakened. In SSP5‐8.5, the fossil fuel emissions are large enough that the fossil fuel contribution to the δ^13^CO_2_ trend remains dominant.

The simulated future changes in atmospheric δ^13^CO_2_ and Δ^14^CO_2_ span a larger range than previous atmospheric carbon isotope studies (Graven, 2015; Köhler, 2016). This is expected because the SSP‐based scenarios span a larger range in atmospheric CO_2_ concentration and fossil fuel emissions than the RCPs. The lowest simulated Δ^14^CO_2_ in 2100 for SSP5‐8.5 is nearly −300‰ while the highest simulated Δ^14^CO_2_ in 2100 for SSP1‐1.9 is above 0‰. The range in the RCPs was −250‰ to −20‰ (Graven, 2015). For δ^13^CO_2_, the lowest simulated value in 2100 for SSP5‐8.5 is nearly −13‰, while the highest simulated value in 2100 for SSP1‐1.9 is approximately 7‰, similar to what it was in 1950. It is difficult to compare these values with Köhler (2016) because his simulations underestimated δ^13^CO_2_ observed over the Industrial Period.

We emphasize that these simulations do not account for all climate change‐related feedbacks to ^13^C and ^14^C fluxes. They do account for temperature‐driven changes to solubility and fractionation that affect air‐sea exchanges, but not for other potential changes to ocean or terrestrial biospheric fluxes. For example, Khatiwala et al. (2018) found that simulated changes in ocean circulation affected the air‐sea ^14^C fluxes over the 21st century, although these fluxes were still within the range simulated by Graven (2015). Changes to ocean circulation could also affect ^13^C fluxes, and other changes such as wind speed not considered by Khatiwala et al. (2018) could affect both ^13^C and ^14^C fluxes through the impact on gas exchange. On land, changes in climate could affect photosynthesis, turnover of biospheric carbon, and permafrost stability with impacts on ^13^C and ^14^C fluxes. However, we do expect that the SSP‐driven changes in emissions and atmospheric CO_2_ concentration that are included in these simulations will be the dominant influences over this century.

## 9 Impacts of Predicted Future Changes

The predicted changes in atmospheric δ^13^CO_2_ and Δ^14^CO_2_ have impacts on the way δ^13^CO_2_ and Δ^14^CO_2_ are used in carbon cycle science and other fields. As described in Graven (2015), high‐emission scenarios that cause strong decreases in Δ^14^CO_2_ provide a continuing atmospheric perturbation that can be tracked to study exchange rates and residence times in different carbon pools. However, these scenarios create problems for applications such as radiocarbon dating because recently produced materials will have the same radiocarbon content as materials produced at some point up to 2,500 years in the past. Figure 6 includes on the right axis the equivalent conventional radiocarbon age. This shows the age of materials with the same ratio of ^14^C/C but where the ratio has been reduced because of radioactive decay rather than dilution by fossil carbon. The “age” of the atmosphere in the highest emission scenario is up to 2,500 years in the year 2100, older than for the highest RCP (Graven, 2015). In this scenario, radiocarbon dating would not be able to distinguish newly produced materials from those up to 2,500 years old by the end of this century, and by 2050, radiocarbon dating would give ambiguous results for samples up to nearly 1,500 years old. These periods encompass much of the development of human civilization when radiocarbon dating has been a key tool in archaeology.

Similarly, applications for forgery detection or illegal ivory trading will be affected because newly produced materials will not be so easily distinguished from older ones. Radiocarbon measurements have been used to date the age of ivory (Cerling et al., 2016), with low radiocarbon content below Modern reflecting ivory produced prior to the 1950s that is not subject to legal restrictions or bans. But new ivory will soon also measure below 0‰, eliminating the use of ^14^C as a detection tool for illegal ivory. Within carbon cycle science, the high‐emission scenarios reduce the effectiveness of using Δ^14^CO_2_ to quantify fossil fuel emissions because the sensitivity of Δ^14^CO_2_ to fossil fuel CO_2_ goes down from −2.6‰ ppm^−1^ presently to −1.6‰ ppm^−1^ in 2050 for high‐emission scenarios (Graven, 2015). Advances in measurement precision are needed to maintain the detection limit for fossil fuel CO_2_, but measurement precision has not improved over the last 10 years.

On the other hand, low‐emission scenarios reduce the impact to these applications above but create different challenges for other applications. Low‐emission scenarios cause Δ^14^CO_2_ to stabilize in the middle to late 21st century, eliminating the temporal change in Δ^14^CO_2_ that formed the basis of many applications examining exchange rates and residence times, both in carbon cycle science and other field such as physiology. For example, in physiology the production of different types of cells can be assessed with their radiocarbon content. The age of a person can be matched to the atmospheric “bomb curve” (Figure 3) to determine what the Δ^14^C in the cells of interest would be at birth or early in life, and then by comparing the Δ^14^C in cells of adults their production rate be estimated (Spalding et al., 2005). If atmospheric Δ^14^CO_2_ stabilizes, then this application cannot be used because the difference in radiocarbon content of materials produced in different years or decades would drop to very low levels. This type of application is now widely used to examine decadal‐scale carbon turnover in soil science (Trumbore, 2000), and it would be difficult to replace with other methods.

For δ^13^CO_2_, the predicted changes also have impacts on applications using atmospheric δ^13^CO_2_ measurements. As atmospheric δ^13^CO_2_ changes, the disequilibrium between atmospheric CO_2_ and the carbon in the terrestrial biosphere and the ocean will also change. Following a low‐emission scenario will result in the atmospheric δ^13^CO_2_ trend reversing and the disequilibrium changing sign. For atmospheric inversions interpreting atmospheric δ^13^CO_2_, it will be important to accurately estimate the changing disequilibrium flux despite potentially complex changes. Studies of plant activity using tree rings could also be complicated by the reversal of the atmospheric δ^13^CO_2_ trend in the low‐emission scenarios, or by the predicted changes in discrimination of several per mil in the high‐emission scenarios (Figure 5). Ocean observations of δ^13^C will also show a more complicated relationship with anthropogenic CO_2_ in the low‐emission scenarios, such that it may not be possible to use ocean δ^13^C data to estimate ocean CO_2_ uptake as it has been used in the past (Gruber & Keeling, 2001; Quay et al., 2003).

## 10 Current Status and Future Needs for Observations and Modeling of Carbon Isotopes

Observations of δ^13^C and Δ^14^C in atmospheric CO_2_ and other carbon reservoirs have enabled important insights on the carbon cycle and on atmospheric and oceanic circulation, as outlined above. The observations from the unique period of the nuclear bomb testing were particularly powerful, and scientific research would have benefitted greatly if an even larger number of observations had been made during that time, across a larger variety of environments, including additional measurements of the atmosphere and ocean as well as the carbon in soils, rivers, and lakes. The geochemist Wally Broecker, who pioneered many radiocarbon applications, used to say, “Instead of publishing papers, we should have just dropped everything and collected samples all over the world.”

Another critical period is now upon us, as Δ^14^CO_2_ drops below 0‰ and either stabilizes or continues dropping to very low levels. Simulations of future atmospheric changes demonstrate that it is unavoidable that some applications for Δ^14^C, and possibly δ^13^C, will become less effective in the future. The specific applications that will be affected depend on the emissions pathway followed. Since the utility of at least some applications is decreasing over time, observations made now will, in general, be more useful than those that will be made in the future. For example, the use of Δ^14^C measurements to establish the decadal‐scale turnover of terrestrial carbon pools will disappear in the future if Δ^14^CO_2_ stabilizes. Therefore, it would be immensely valuable to make concerted, coordinated efforts to conduct more observations of Δ^14^C in the Earth System as soon as possible. The sooner we make the observations, the more we will achieve with them.

Currently, observations of atmospheric δ^13^CO_2_ and Δ^14^CO_2_ are conducted by several laboratories operating global or regional networks of stations. Global networks for δ^13^CO_2_ are operated by the US National Oceanic and Atmospheric Administration (NOAA), Australia's Commonwealth Scientific and Industrial Research Organisation (CSIRO), and Scripps Institution of Oceanography (SIO). Only one global network for Δ^14^CO_2_ is currently being operated, by the University of Heidelberg, although other global networks have operated in the past (Graven et al., 2012a; Nydal & Lövseth, 1983). There are regional networks for Δ^14^CO_2_ and δ^13^CO_2_ in Europe as part of the Integrated Carbon Observing System (ICOS) and in North America by NOAA and other laboratories. Urban‐scale networks have also been developed (Turnbull et al., 2015). Most of these observations are publicly available, for example, through the World Data Centre for Greenhouse Gases (https://gaw.kishou.go.jp/) or the ICOS portal (https://www.icos‐cp.eu/).

Little is known about the current atmospheric distribution of Δ^14^CO_2_ and δ^13^CO_2_ away from the surface. There have been some stratospheric observations of Δ^14^CO_2_ conducted since the late 1980s (Kanu et al., 2016; Nakamura et al., 1994), but these comprise only a handful of vertical profiles. NOAA conducts regular aircraft measurements of Δ^14^CO_2_ in the troposphere at some sites in North America (Estevan Point, Park Falls, Cape May, and Portsmouth) and at a larger network of sites for δ^13^CO_2_ (Miller et al., 2012; Sweeney et al., 2015). Some other aircraft measurements of δ^13^CO_2_ have also been made, showing influences of biospheric exchange and atmospheric mixing in the northern free troposphere and influences of the stratosphere in the tropopause region (Assonov et al., 2010; Levin et al., 2002). More observations from aircraft would help to refine our understanding of δ^13^CO_2_ and Δ^14^CO_2_ variations through the atmosphere, with applications for assessing biospheric fluxes, fossil fuel emissions, and atmospheric transport. The implementation of laboratory calibration recommendations and continued intercomparison activities are needed to ensure that data from different labs can be combined (WMO/IAEA, 2016).

In addition to efforts expanding the observations of Δ^14^C and δ^13^C across the carbon cycle, efforts to make modeling tools more openly available are needed to optimize the scientific advances that can be made with Δ^14^C and δ^13^C observations. We believe that existing observations are underutilized at present because isotopic modeling tools and expertise are not widely available or widely used. Modeling of atmospheric δ^13^CO_2_ and Δ^14^CO_2_ is typically done on a case‐by‐case basis. There is currently a lack of shared atmospheric modeling tools for isotopic simulations. Models are used on global scales and on regional scales, ranging from box models to high‐resolution three‐dimensional transport models (Basu et al., 2016; Keeling et al., 2017; Peters et al., 2018). To simulate atmospheric δ^13^CO_2_ and Δ^14^CO_2_, models or data‐based estimates of the carbon and isotopic fluxes from relevant processes are also needed. Here too, various models and estimates of isotopic fluxes have been used in individual studies, but not many of these are made available for other researchers. To provide modeled isotopic fluxes for use by the community, and to promote isotopic modeling in general, it was recommended that modeling groups in the latest Coupled Model Intercomparison Project activity (CMIP6) simulate carbon isotopes in the land and ocean modules of their Earth System Models using a specified atmospheric boundary condition (Figure 3, Graven et al., 2017; Jones et al., 2016; Orr et al., 2017). Only one model, CESM2, has so far included carbon isotopes in their CMIP6 simulations. It is hoped that the next phase of the CMIP will include more isotopic modeling and that isotopic modeling will be incorporated in other large modeling activities. Simulation of atmospheric δ^13^CO_2_ and Δ^14^CO_2_ in the atmospheric models of Earth System Models has not yet been implemented, so it is currently not possible to do a fully coupled simulation of δ^13^C and Δ^14^C using the most state‐of‐the‐art models. Such fully coupled isotopic models would be useful not only for the modern period but also for paleoclimate modeling. Other shared tools enabling atmospheric modeling of δ^13^CO_2_ and Δ^14^CO_2_ would also help to exploit existing and future atmospheric measurements.

To fully develop the use of Δ^14^CO_2_ observations to monitor regional emissions from fossil fuel combustion, many more observations and better modeling capabilities on regional scales are needed. Studies of regional atmospheric measurement networks combined with high‐resolution atmospheric modeling have only recently been published (Basu et al., 2020; Graven et al., 2018), and best practices are still under development. For example, different studies have constructed atmospheric inversions differently. The methods used in Graven et al. (2018) and Fischer et al. (2017) first calculate fossil fuel‐derived CO_2_ (ffCO_2_, Equation 1) and biospheric CO_2_ and then run an inversion for fossil fuel and biospheric fluxes. In contrast, Basu et al. (2016) set up their inversion to estimate individual ^14^CO_2_ and CO_2_ fluxes across North America, including all the processes that can influence Δ^14^CO_2_ in the inversion. Other best practices that are still under development include the location and sampling height of observation sites in the network. Sites that have lower sampling heights or that are located closer to emission sources have higher signals in ffCO_2_, whereas sites with higher sampling heights or located further from sources have lower signals, but they represent larger regions. Having more than one observation site within a particular region can be important to prevent biases from any unique site characteristics, for example, including both urban and ex‐urban sites that differ not only in their ffCO_2_ signals or representation scale but also in the atmospheric model's representation of the transport in different types of regions (Brophy et al., 2019). In some locations, the ^14^C emissions from nuclear power plants can cause enrichment of Δ^14^CO_2_, particularly near to high ^14^C‐emitting reactors in the United Kingdom and Canada (Bozhinova et al., 2014; Graven & Gruber, 2011; Vogel et al., 2013). A better understanding of ^14^C emissions from nuclear power plants and better ^14^C emissions data would enable their effect to be accurately accounted for and improve the utility of Δ^14^CO_2_ for ffCO_2_ quantification in regions with nuclear power plants. Further development of regional networks for Δ^14^CO_2_ and complementary measurements including satellite observations, as well as the model‐data analysis frameworks for interpreting the observations to constrain ffCO_2_ emissions, are needed (Ciais et al., 2015; Fischer et al., 2017).

## 11 Summary

Since the Industrial Revolution, the carbon isotopic composition of atmospheric CO_2_ has undergone dramatic changes as a result of human activities and the response of the natural carbon cycle to them. The relative amount of atmospheric ^14^C and ^13^C in CO_2_ has decreased because of the addition of ^14^C‐ and ^13^C‐depleted fossil carbon, while the nuclear bomb tests increased ^14^C in the atmosphere in the 1950s and 1960s. Measurements of Δ^14^CO_2_ and δ^13^CO_2_ have been used to make invaluable contributions to our knowledge of atmospheric mixing, air‐sea gas exchange, plant function, and fossil fuel emissions. As fossil fuel burning continues to grow, the Suess Effect on ^14^C and ^13^C in CO_2_ continues. However, lower‐emission scenarios would lead to stabilized Δ^14^CO_2_ and increases in δ^13^CO_2_ over this century. The different paths described by the SSP‐based scenarios show that there is a wide range of possible changes to ^14^C and ^13^C of CO_2_ in the future. Researchers should be aware of the possible changes and their effect on the continued utility of ^14^C and ^13^C measurements of CO_2_ for scientific applications across various fields. We recommend a concerted effort to increase the number of ^14^C and ^13^C measurements across the Earth System and more development of publicly available modeling tools that incorporate ^14^C and ^13^C, including Earth System Models.

## Supporting information

Supporting Information S1

Table S1

Table S2

## Data Availability

Historical and future atmospheric forcing data sets for Δ^14^CO_2_ and δ^13^CO_2_ can be accessed at input4MIPs (https://esgf‐node.llnl.gov/search/input4mips/). The future Δ^14^CO_2_ and δ^13^CO_2_ data sets are also given in Table S2. SSP‐based emissions scenarios are hosted by the International Institute for Applied Systems Analysis and available online (from https://tntcat.iiasa.ac.at/SspDb/). The simple carbon cycle model is available online (at https://github.com/heathergraven/simplemodel2020).
