# Supplementary material for: Changes to Carbon Isotopes in Atmospheric CO2 Over the Industrial Era and Into the Future
Source: Global Biogeochem Cycles. 2020 Nov 15;34(11):e2019GB006170. doi: 10.1029/2019GB006170 (PMC7757245; doi:10.1029/2019GB006170)
Supplement: Supplementary file 1 — Supporting Information S1 [file GBC-34-e2019GB006170-s001.pdf]

Global Biogeochemical Cycles

Supporting Information for

Changes to Carbon Isotopes in Atmospheric CO_2 over the Industrial Era and into the Future

Heather Graven[1,2], Ralph F. Keeling[3] and Joeri Rogelj[2,4]

[1]Department of Physics, Imperial College London, London, UK.
[2]Grantham Institute for Climate Change and the Environment, Imperial College London, London, UK.
[3]Scripps Institution of Oceanography, University of California, San Diego, USA.
[4]ENE Program, International Institute for Applied Systems Analysis, Laxenburg, Austria.

Contents of this file

Text SM1 and SM2
Figure S1
Table S1
Table S2

Text SM1. Simulations for “no bombs” and “no fossil” scenarios

Simulations for $\Delta^{14}C$ and $\delta^{13}C$ of atmospheric CO_2 were conducted following Graven (2015). The model has one atmosphere box and 43 ocean boxes in a one-dimensional box diffusion model. Graven (2015) used a one-box terrestrial biosphere but here we have changed the model so that the biosphere is represented by three boxes representing fast, medium and slow turnover pools. There is no representation of carbon transport from terrestrial boxes to ocean boxes via rivers.

To run the simulation with no ^{14}C from nuclear weapons tests, atmospheric CO_2 concentration and $\Delta^{14}CO_2$ are computed prognostically, accounting for fossil fuel emissions and natural ^{14}C production. To run the simulation with no fossil fuel emissions, the input of ^{14}C from the nuclear weapons tests that is consistent with the observations, within the structure of the simple model, is first diagnosed with a historical run forced

with observed atmospheric $\Delta^{14}CO_2$. The annual change in total ^{14}C inventory of all the carbon pools in this run is then specified as the ^{14}C from the nuclear weapons tests. The total production is 634 x10^{26} atoms through 1980, which is comparable to the estimate of *Naegler and Levin* [2006]: 598–632 x 10^{26} atoms between 1945 and 1980, both including ^{14}C production by the nuclear power industry. Then a simulation is run with the specified annual input of ^{14}C from nuclear weapons tests from the previous simulation through 2005 and then with constant ^{14}C production from natural and anthropogenic sources for 2005-2015, but with no fossil fuel emissions. Land use change emissions are included in both simulations. For both simulations, the model is run with one set of parameters (Table S1) chosen from the sets of calibrated parameters as described in the next section.

Text SM2. Simulations for SSP-based scenarios

Simulations for $\Delta^{14}C$ and $\delta^{13}C$ of atmospheric CO_2 were conducted following *Graven* [2015].

Simulations were conducted for scenarios that are based on the SSP framework, the four Tier 1 ScenarioMIP scenarios (SSP1-2.6, SSP2-4.5, SSP3-7.0, and SSP5-8.5) and two of the Tier 2 scenarios (SSP1-1.9 SSP5-3.4-Overshoot) [*O'Neill et al.*, 2016]. These six scenarios span the full range of radiative forcing and CO_2 emissions included in the larger set of SSP-based scenarios.

The simulations use historical data and data for the six SSP-based scenarios. Historical atmospheric CO_2 concentration was specified by global annual mean data from *Meinshausen et al.* [2017]. Historical fossil fuel CO_2 emissions data were given by the Community Emissions Data System (CEDS) [*Hoesly et al.*, 2018]. Historical land use CO_2 emissions data were given by C4MIP (CMIP6_C4MIP_landuse_emissions.nc available at http://c4mip.net/index.php?id=3455). Global annual atmospheric CO_2 concentration data for the SSP scenarios were retrieved from input4mips (https://esgf-node.llnl.gov/projects/input4mips/). The SSP fossil fuel and land use CO_2 emissions and the SSP bioenergy with carbon capture and storage (BECCS) data were gathered from the SSP database hosted at the International Institute for Applied Systems Analysis (https://tntcat.iiasa.ac.at/SspDb/), and accessed in October 2018.

The $\delta^{13}C$ of fossil fuel emissions was specified by *Andres et al.* [2016] for 1850 to 2013 and then kept fixed at the 2013 value from *Andres et al.* [2016], -27.73 per mil, for the period 2013 to 2100 in all SSPs. Due to limitations in the data available about the SSP-based scenarios it was not possible to accurately specify $\delta^{13}C$ in fossil fuel emissions in the scenario projections, i.e. CO_2 emissions or time-varying emission factors (amount of CO_2 emitted per unit energy produced) for each fuel type were not reported in the SSP database, despite each fuel type having a specific ^{13}C fingerprint. Sensitivity tests estimating future changes in $\delta^{13}C$ of fossil fuel emissions showed that the potential

impact on simulated atmospheric $\delta^{13}C$ is likely to be small, less than ~0.4 per mil for simulated $\delta^{13}C$ in 2100.

Simulations for 1850-2005 were conducted following a spinup period of 11850 years. Atmospheric $\Delta^{14}C$ and atmospheric $\delta^{13}C$ were specified by historical data from *Graven et al.* [2017] for 1850-2005 and then atmospheric $\Delta^{14}CO_2$ and $\delta^{13}CO_2$ were simulated prognostically for 2005-2100.

Three changes were made to the model setup in comparison to *Graven* [2015].

First, a sensitivity of photosynthetic ^{13}C discrimination to atmospheric CO_2 concentration was included, following *Schubert and Jahren* [2015] and *Keeling et al.* [2017]. The formulation for discrimination in *Schubert and Jahren* [2015]'s equations 3 and 4 was used, beginning from ^{13}C discrimination of 17 per mil in 1850. This leads to discrimination of 18.7 per mil in 2100 for SSP1-1.9 and 22.5 per mil in 2100 for SSP5-8.5.

Second, sea surface temperature (SST) changes were included for both the historical and future period. The historical data used were annual, global data SST anomalies from HadSST.3.1.1.0 from 1850 to 2018 (https://www.metoffice.gov.uk/hadobs/hadsst3/data/download.html, accessed October 2018). A reference SST of 18°C in 1850 was assumed. Future SST was estimated from global mean temperature data for the SSPs retrieved from the internal SSP database. SST anomalies in the SSPs for 2020 to 2100 were estimated from global mean temperature by applying a scaling factor of 0.85, which derives from a regression of HadSST.3.1.1.0 SST anomaly data and HadCRUT.4.6.0.0 global annual near surface temperature anomaly data (https://www.metoffice.gov.uk/hadobs/hadcrut4/data/current/download.html, accessed October 2018). SST is used in the model in the calculation of the ocean carbonate system and in the specification of fractionation factors related to air-sea gas exchange.

Third, the formulation of the terrestrial biosphere model was changed from one to three boxes, following *Naegler and Levin* [2009]. The initial masses and turnover times in the scenarios listed in *Naegler and Levin* [2009]'s Table 1 were investigated with other parameters in the model following the parameter ranges used in *Graven* [2015]. Parameter sets were selected to match a global bomb excess ^{14}C inventory of $615 \pm 35 \times 10^{26}$ atoms and to match the change in atmospheric $\delta^{13}C$ between 2005 and 2014 to within ±0.05 per mil, resulting in 34 different accepted parameter sets (Table S1). The mid-range values for simulated atmospheric $\Delta^{14}C$ and atmospheric $\delta^{13}C$ across the accepted parameter sets are used to specify the forcing for SSP-based projections.

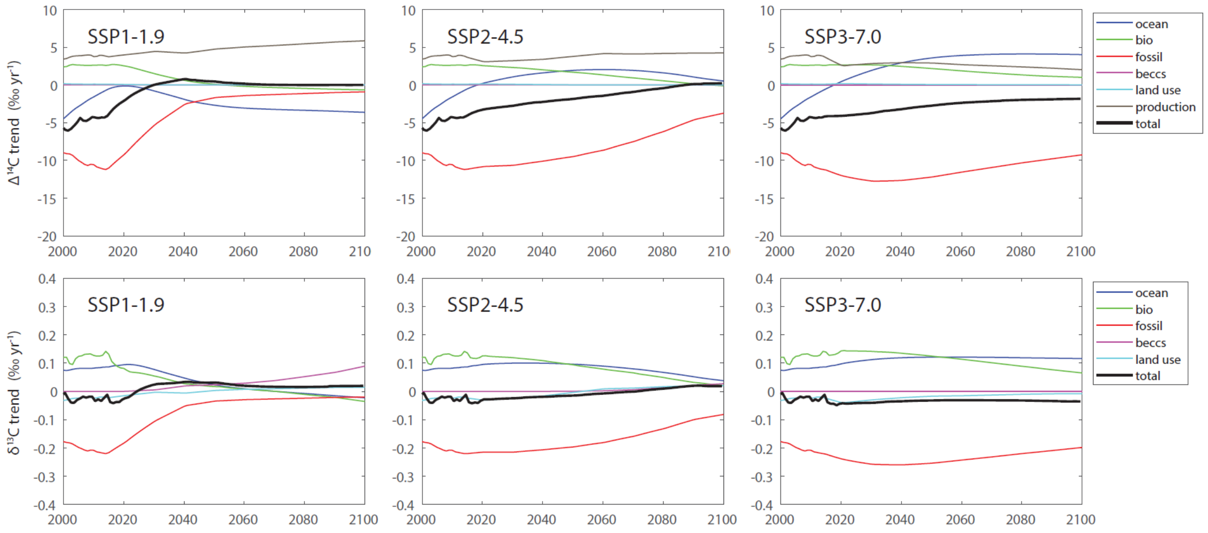

Figure S1. Simulated trend components for $\Delta^{14}CO_2$ (top row) and $\delta^{13}CO_2$ (bottom row) for SSP1-1.9, SSP2-4.5 and SSP3-7.0. Colored lines show the mid-range values across the 32 sets of parameters used in the simulations. BECCS and land use contributions are uniformly near zero for $\Delta^{14}CO_2$.

Table S1. Calibrated parameter sets used in the future simulations. Parameters are as described in *Graven* [2015] but here there are three biosphere boxes instead of one. The units for Keddy are m2 yr-1 and units for all the τ's are years. β is unitless. The parameter set in the first row was used in the simulations excluding fossil fuel emissions or nuclear weapons testing shown in Figure 4.

Keddy, $m^2\ y^{-1}$	τam	β	τab1	τab2	τab3	τba1	τba2	τba3
4000	10	0.4	27.1	21.6	210.9	2.4	24.8	580.2
4000	10	0.4	33.8	25.6	61.9	2	13.4	212.8
4000	11	0.4	21.2	31.5	53.0	2.6	24.1	165.7
3000	9	0.4	33.8	25.6	61.9	2	13.4	212.8
3000	10	0.4	33.8	25.6	61.9	2	13.4	212.8
3000	11	0.4	33.8	25.6	61.9	2	13.4	212.8
3000	11	0.4	21.2	31.5	53.0	2.6	24.1	165.7
4000	9	0.4	38.1	23.0	107.8	2	15.7	353.2
3000	9	0.4	38.1	23.0	107.8	2	15.7	353.2
5000	11	0.4	23.2	24.4	105.4	2.5	26.2	299.4
5000	10	0	33.8	25.6	61.9	2	13.4	212.8
4000	11	0.4	23.2	24.4	105.4	2.5	26.2	299.4
4000	10	0	33.8	25.6	61.9	2	13.4	212.8
4000	11	0	33.8	25.6	61.9	2	13.4	212.8
3000	10	0.4	23.2	24.4	105.4	2.5	26.2	299.4

3000	11	0.4	23.2	24.4	105.4	2.5	26.2	299.4
3000	9	0	33.8	25.6	61.9	2	13.4	212.8
3000	10	0	33.8	25.6	61.9	2	13.4	212.8
3000	11	0	33.8	25.6	61.9	2	13.4	212.8
4000	9	0	38.1	23.0	107.8	2	15.7	353.2
4000	11	0	21.2	31.5	53.0	2.6	24.1	165.7
3000	9	0	38.1	23.0	107.8	2	15.7	353.2
3000	11	0	21.2	31.5	53.0	2.6	24.1	165.7
3000	9	0.4	27.1	21.6	210.9	2.4	24.8	580.2
3000	10	0.4	27.1	21.6	210.9	2.4	24.8	580.2
5000	11	0	23.2	24.4	105.4	2.5	26.2	299.4
4000	11	0	23.2	24.4	105.4	2.5	26.2	299.4
4000	10	0	27.1	21.6	210.9	2.4	24.8	580.2
3000	10	0	23.2	24.4	105.4	2.5	26.2	299.4
3000	11	0	23.2	24.4	105.4	2.5	26.2	299.4
3000	9	0	27.1	21.6	210.9	2.4	24.8	580.2
3000	10	0	27.1	21.6	210.9	2.4	24.8	580.2
3000	11	0.4	21.7	20.1	232.6	2.6	34.7	502.4
3000	11	0	21.7	20.1	232.6	2.6	34.7	502.4

Table S2. Atmospheric forcing datasets for $\Delta^{14}CO_2$ and $\delta^{13}CO_2$ for the six SSP-based scenarios. Units are ‰.

	1-1.9	1-1.9	1-2.6	1-2.6	2-4.5	2-4.5	3-7.0	3-7.0	5-3.4os	5-3.4os	5-8.5	5-8.5
	$\Delta^{14}C$	$\delta^{13}C$	$\Delta^{14}C$	$\delta^{13}C$	$\Delta^{14}C$	$\delta^{13}C$	$\Delta^{14}C$	$\delta^{13}C$	$\Delta^{14}C$	$\delta^{13}C$	$\Delta^{14}C$	$\delta^{13}C$
2005	66.1	-8.20	66.1	-8.20	66.1	-8.20	66.1	-8.20	66.1	-8.20	66.1	-8.20
2006	61.7	-8.22	61.7	-8.22	61.7	-8.22	61.7	-8.22	61.7	-8.22	61.7	-8.22
2007	57.0	-8.24	57.0	-8.24	57.0	-8.24	57.0	-8.24	57.0	-8.24	57.0	-8.24
2008	52.2	-8.26	52.2	-8.26	52.2	-8.26	52.2	-8.26	52.2	-8.26	52.2	-8.26
2009	47.6	-8.28	47.6	-8.28	47.6	-8.28	47.6	-8.28	47.6	-8.28	47.6	-8.28
2010	43.4	-8.30	43.4	-8.30	43.4	-8.30	43.4	-8.30	43.4	-8.30	43.4	-8.30
2011	39.1	-8.34	39.1	-8.34	39.1	-8.34	39.1	-8.34	39.1	-8.34	39.1	-8.34
2012	34.7	-8.36	34.7	-8.36	34.7	-8.36	34.7	-8.36	34.7	-8.36	34.7	-8.36
2013	30.4	-8.40	30.4	-8.40	30.4	-8.40	30.4	-8.40	30.4	-8.40	30.4	-8.40
2014	26.1	-8.42	26.1	-8.42	26.1	-8.42	26.1	-8.42	26.1	-8.42	26.1	-8.42
2015	21.9	-8.43	21.9	-8.43	21.9	-8.43	21.9	-8.43	21.9	-8.43	21.9	-8.43
2016	18.3	-8.47	18.0	-8.47	18.0	-8.47	17.7	-8.47	17.8	-8.47	17.8	-8.47
2017	15.1	-8.51	14.4	-8.51	14.2	-8.51	13.6	-8.51	13.8	-8.51	13.8	-8.51
2018	12.3	-8.55	10.9	-8.55	10.7	-8.55	9.5	-8.56	9.9	-8.55	9.9	-8.55
2019	9.8	-8.59	7.6	-8.59	7.2	-8.59	5.3	-8.61	6.0	-8.60	6.0	-8.60
2020	7.6	-8.61	4.4	-8.62	3.9	-8.62	1.2	-8.65	2.1	-8.64	2.1	-8.64
2021	5.6	-8.64	1.3	-8.64	0.7	-8.65	-2.9	-8.69	-1.7	-8.68	-1.7	-8.68

2022	3.9	-8.66	-1.6	-8.67	-2.5	-8.68	-6.9	-8.73	-5.6	-8.72	-5.6	-8.71
2023	2.5	-8.67	-4.3	-8.69	-5.6	-8.71	-10.9	-8.78	-9.6	-8.76	-9.6	-8.75
2024	1.4	-8.68	-6.9	-8.71	-8.7	-8.73	-14.9	-8.82	-13.6	-8.81	-13.6	-8.80
2025	0.5	-8.68	-9.4	-8.72	-11.7	-8.76	-18.9	-8.86	-17.7	-8.85	-17.7	-8.84
2026	-0.3	-8.67	-11.8	-8.73	-14.7	-8.79	-22.8	-8.90	-21.8	-8.90	-21.8	-8.89
2027	-0.8	-8.66	-14.0	-8.74	-17.6	-8.81	-26.7	-8.94	-25.9	-8.94	-25.9	-8.93
2028	-1.2	-8.64	-16.2	-8.75	-20.5	-8.84	-30.5	-8.98	-30.0	-8.99	-30.0	-8.98
2029	-1.4	-8.62	-18.3	-8.76	-23.3	-8.86	-34.3	-9.02	-34.1	-9.04	-34.2	-9.03
2030	-1.5	-8.60	-20.3	-8.76	-26.1	-8.89	-38.1	-9.06	-38.2	-9.09	-38.3	-9.07
2031	-1.4	-8.58	-22.2	-8.77	-28.8	-8.91	-41.8	-9.10	-42.4	-9.14	-42.5	-9.12
2032	-1.2	-8.55	-24.0	-8.77	-31.5	-8.94	-45.4	-9.14	-46.6	-9.18	-46.7	-9.17
2033	-1.0	-8.52	-25.5	-8.77	-34.1	-8.96	-49.0	-9.18	-50.8	-9.23	-50.9	-9.22
2034	-0.7	-8.50	-26.9	-8.77	-36.6	-8.98	-52.5	-9.22	-55.1	-9.28	-55.2	-9.27
2035	-0.4	-8.47	-28.2	-8.76	-39.1	-9.00	-55.9	-9.26	-59.4	-9.34	-59.5	-9.32
2036	0.1	-8.44	-29.3	-8.76	-41.5	-9.02	-59.4	-9.29	-63.7	-9.39	-63.8	-9.37
2037	0.6	-8.41	-30.3	-8.75	-43.9	-9.04	-62.7	-9.33	-68.1	-9.44	-68.2	-9.42
2038	1.2	-8.38	-31.3	-8.75	-46.2	-9.06	-66.1	-9.36	-72.5	-9.49	-72.6	-9.47
2039	1.9	-8.35	-32.1	-8.74	-48.5	-9.08	-69.4	-9.40	-76.9	-9.54	-77.0	-9.53
2040	2.6	-8.31	-32.8	-8.73	-50.8	-9.10	-72.6	-9.43	-81.2	-9.60	-81.4	-9.58
2041	3.4	-8.28	-33.5	-8.72	-53.0	-9.12	-75.8	-9.47	-85.5	-9.65	-85.8	-9.63
2042	4.1	-8.25	-34.1	-8.71	-55.2	-9.14	-78.9	-9.50	-89.2	-9.70	-90.2	-9.68
2043	4.8	-8.21	-34.7	-8.69	-57.3	-9.16	-82.0	-9.54	-92.4	-9.74	-94.6	-9.74
2044	5.4	-8.18	-35.2	-8.68	-59.4	-9.17	-85.0	-9.57	-95.1	-9.77	-99.0	-9.79
2045	6.0	-8.15	-35.7	-8.67	-61.4	-9.19	-88.0	-9.60	-97.4	-9.80	-103.4	-9.85
2046	6.6	-8.12	-36.1	-8.65	-63.5	-9.21	-90.9	-9.64	-99.4	-9.81	-107.8	-9.90
2047	7.1	-8.09	-36.4	-8.64	-65.4	-9.22	-93.8	-9.67	-101.0	-9.82	-112.2	-9.96
2048	7.6	-8.06	-36.8	-8.62	-67.4	-9.24	-96.6	-9.70	-102.5	-9.83	-116.6	-10.01
2049	8.1	-8.03	-37.0	-8.60	-69.3	-9.25	-99.4	-9.73	-103.7	-9.83	-121.0	-10.07
2050	8.5	-8.00	-37.3	-8.59	-71.2	-9.27	-102.2	-9.76	-104.7	-9.82	-125.3	-10.12
2051	9.0	-7.97	-37.5	-8.57	-73.0	-9.28	-104.9	-9.80	-105.4	-9.82	-129.7	-10.18
2052	9.4	-7.93	-37.6	-8.55	-74.8	-9.30	-107.6	-9.83	-105.9	-9.80	-134.0	-10.23
2053	9.7	-7.91	-37.8	-8.53	-76.5	-9.31	-110.3	-9.86	-106.1	-9.79	-138.4	-10.29
2054	10.0	-7.88	-37.9	-8.51	-78.2	-9.32	-112.8	-9.89	-106.0	-9.76	-142.7	-10.35
2055	10.3	-7.85	-38.0	-8.49	-79.8	-9.33	-115.4	-9.92	-105.7	-9.73	-147.1	-10.41
2056	10.5	-7.83	-38.1	-8.47	-81.4	-9.34	-117.9	-9.95	-105.2	-9.69	-151.5	-10.46
2057	10.8	-7.80	-38.1	-8.45	-83.0	-9.35	-120.4	-9.98	-104.5	-9.65	-155.8	-10.52
2058	11.0	-7.78	-38.1	-8.43	-84.5	-9.36	-122.8	-10.02	-103.7	-9.61	-160.2	-10.58
2059	11.2	-7.76	-38.1	-8.41	-86.0	-9.37	-125.3	-10.05	-102.6	-9.56	-164.5	-10.64
2060	11.4	-7.74	-38.1	-8.39	-87.4	-9.38	-127.6	-10.08	-101.4	-9.50	-168.8	-10.70
2061	11.5	-7.72	-38.1	-8.37	-88.9	-9.38	-130.0	-10.11	-99.9	-9.44	-173.0	-10.76
2062	11.6	-7.70	-38.1	-8.35	-90.2	-9.39	-132.3	-10.14	-98.5	-9.39	-177.2	-10.82
2063	11.8	-7.68	-38.0	-8.33	-91.5	-9.40	-134.6	-10.17	-97.0	-9.32	-181.4	-10.88
2064	11.9	-7.66	-38.0	-8.31	-92.8	-9.40	-136.9	-10.20	-95.5	-9.26	-185.5	-10.94

2065	11.9	-7.65	-37.9	-8.29	-94.0	-9.40	-139.2	-10.23	-94.0	-9.20	-189.5	-11.00
2066	12.0	-7.63	-37.8	-8.27	-95.1	-9.41	-141.4	-10.27	-92.5	-9.14	-193.5	-11.06
2067	12.0	-7.61	-37.7	-8.25	-96.2	-9.41	-143.7	-10.30	-90.9	-9.07	-197.5	-11.12
2068	12.1	-7.59	-37.5	-8.23	-97.2	-9.41	-145.9	-10.33	-89.3	-9.01	-201.4	-11.18
2069	12.1	-7.58	-37.4	-8.21	-98.2	-9.41	-148.1	-10.36	-87.6	-8.94	-205.3	-11.24
2070	12.1	-7.56	-37.2	-8.19	-99.2	-9.42	-150.2	-10.39	-85.9	-8.87	-209.1	-11.30
2071	12.2	-7.55	-37.1	-8.17	-100.1	-9.42	-152.4	-10.42	-84.1	-8.80	-212.9	-11.36
2072	12.2	-7.53	-36.9	-8.15	-100.9	-9.42	-154.5	-10.45	-82.4	-8.73	-216.6	-11.43
2073	12.2	-7.51	-36.7	-8.13	-101.7	-9.41	-156.6	-10.48	-80.7	-8.67	-220.2	-11.49
2074	12.2	-7.50	-36.4	-8.10	-102.4	-9.41	-158.7	-10.52	-79.2	-8.60	-223.7	-11.54
2075	12.2	-7.48	-36.2	-8.08	-103.1	-9.41	-160.8	-10.55	-77.6	-8.54	-227.2	-11.60
2076	12.2	-7.47	-35.9	-8.06	-103.7	-9.40	-162.8	-10.58	-76.1	-8.48	-230.6	-11.66
2077	12.2	-7.45	-35.6	-8.03	-104.3	-9.39	-164.8	-10.61	-74.6	-8.42	-234.0	-11.72
2078	12.1	-7.43	-35.2	-8.01	-104.8	-9.39	-166.8	-10.64	-73.1	-8.37	-237.3	-11.78
2079	12.1	-7.42	-34.9	-7.98	-105.3	-9.38	-168.8	-10.67	-71.5	-8.31	-240.5	-11.84
2080	12.1	-7.40	-34.5	-7.95	-105.8	-9.37	-170.8	-10.71	-70.0	-8.26	-243.7	-11.90
2081	12.1	-7.39	-34.1	-7.93	-106.2	-9.36	-172.8	-10.74	-68.5	-8.20	-246.8	-11.95
2082	12.1	-7.37	-33.7	-7.90	-106.5	-9.35	-174.8	-10.77	-67.1	-8.15	-249.7	-12.01
2083	12.1	-7.35	-33.4	-7.87	-106.8	-9.33	-176.7	-10.80	-65.7	-8.10	-252.6	-12.07
2084	12.0	-7.34	-33.0	-7.85	-107.0	-9.32	-178.7	-10.84	-64.4	-8.06	-255.3	-12.12
2085	12.0	-7.32	-32.7	-7.82	-107.2	-9.31	-180.6	-10.87	-63.1	-8.01	-258.0	-12.17
2086	12.0	-7.31	-32.3	-7.80	-107.3	-9.29	-182.6	-10.90	-61.9	-7.97	-260.6	-12.22
2087	12.0	-7.29	-32.0	-7.77	-107.3	-9.27	-184.5	-10.94	-60.7	-7.93	-263.1	-12.27
2088	12.0	-7.27	-31.6	-7.75	-107.3	-9.26	-186.4	-10.97	-59.5	-7.89	-265.5	-12.32
2089	12.0	-7.25	-31.3	-7.72	-107.3	-9.24	-188.3	-11.00	-58.3	-7.85	-267.9	-12.37
2090	12.0	-7.24	-30.9	-7.70	-107.2	-9.22	-190.2	-11.04	-57.2	-7.81	-270.3	-12.41
2091	12.0	-7.22	-30.6	-7.67	-107.1	-9.20	-192.1	-11.07	-56.1	-7.78	-272.5	-12.46
2092	12.0	-7.20	-30.3	-7.65	-106.9	-9.18	-194.0	-11.10	-55.1	-7.74	-274.7	-12.50
2093	11.9	-7.18	-30.0	-7.63	-106.8	-9.16	-195.9	-11.14	-54.2	-7.71	-276.8	-12.55
2094	11.9	-7.16	-29.7	-7.61	-106.6	-9.14	-197.8	-11.17	-53.3	-7.67	-278.9	-12.59
2095	11.9	-7.14	-29.5	-7.59	-106.4	-9.12	-199.7	-11.21	-52.5	-7.64	-280.9	-12.63
2096	11.9	-7.12	-29.3	-7.57	-106.2	-9.10	-201.5	-11.24	-51.7	-7.61	-282.9	-12.67
2097	11.9	-7.10	-29.1	-7.56	-106.1	-9.08	-203.4	-11.28	-50.9	-7.58	-284.8	-12.71
2098	11.9	-7.08	-28.9	-7.54	-105.9	-9.06	-205.2	-11.31	-50.2	-7.55	-286.6	-12.75
2099	11.9	-7.06	-28.7	-7.53	-105.6	-9.04	-207.1	-11.35	-49.5	-7.52	-288.4	-12.79
2100	11.9	-7.04	-28.6	-7.51	-105.4	-9.03	-208.9	-11.38	-48.8	-7.49	-290.2	-12.83

References

Andres, R. J., T. A. Boden, and G. Marland (2016), Annual Fossil-Fuel CO2 Emissions: Global Stable Carbon Isotopic Signature, edited, Carbon Dioxide Information Analysis

Center, Oak Ridge National Laboratory, U.S. Department of Energy, Oak Ridge, Tenn., U.S.A.
Graven, H., et al. (2017), Compiled records of carbon isotopes in atmospheric CO2 for historical simulations in CMIP6, Geosci. Model Dev., 10(12), 4405-4417, doi: 10.5194/gmd-10-4405-2017.
Graven, H. D. (2015), Impact of fossil fuel emissions on atmospheric radiocarbon and various applications of radiocarbon over this century, P Natl Acad Sci USA, 112(31), 9542-9545, doi: doi:10.1073/pnas.1504467112.
Hoesly, R. M., et al. (2018), Historical (1750–2014) anthropogenic emissions of reactive gases and aerosols from the Community Emissions Data System (CEDS), Geosci. Model Dev., 11(1), 369-408, doi: 10.5194/gmd-11-369-2018.
Keeling, R. F., H. D. Graven, L. R. Welp, L. Resplandy, J. Bi, S. C. Piper, Y. Sun, A. Bollenbacher, and H. A. J. Meijer (2017), Atmospheric evidence for a global secular increase in carbon isotopic discrimination of land photosynthesis, Proceedings of the National Academy of Sciences, doi: 10.1073/pnas.1619240114.
Meinshausen, M., et al. (2017), Historical greenhouse gas concentrations for climate modelling (CMIP6), Geosci Model Dev, 10(5), 2057-2116, doi: doi:10.5194/gmd-10-2057-2017.
Naegler, T., and I. Levin (2006), Closing the global radiocarbon budget 1945–2005, J Geophys Res, 111(D12), D12311, doi: doi:10.1029/2005jd006758.
Naegler, T., and I. Levin (2009), Biosphere-atmosphere gross carbon exchange flux and the $\delta^{13}CO_2$ and $\Delta^{14}CO_2$ disequilibria constrained by the biospheric excess radiocarbon inventory, J Geophys Res, 114(D17), D17303, doi: doi:10.1029/2008jd011116.
O'Neill, B. C., et al. (2016), The Scenario Model Intercomparison Project (ScenarioMIP) for CMIP6, Geosci. Model Dev., 9(9), 3461-3482, doi: 10.5194/gmd-9-3461-2016.
Schubert, B. A., and A. H. Jahren (2015), Global increase in plant carbon isotope fractionation following the Last Glacial Maximum caused by increase in atmospheric pCO2, Geology, 43(5), 435-438, doi: 10.1130/g36467.1.
